# Principal Component Analysis Characterizes Shared Pathogenetics from Genome-Wide Association Studies

**DOI:** 10.1371/journal.pcbi.1003820

**Published:** 2014-09-11

**Authors:** Diana Chang, Alon Keinan

**Affiliations:** 1 Department of Biological Statistics & Computational Biology, Cornell University, Ithaca, New York, United States of America; 2 Program in Computational Biology and Medicine, Cornell University, Ithaca, New York, United States of America; University of Gothenburg, Sweden

## Abstract

Genome-wide association studies (GWASs) have recently revealed many genetic associations that are shared between different diseases. We propose a method, *disPCA*, for genome-wide characterization of shared and distinct risk factors between and within disease classes. It flips the conventional GWAS paradigm by analyzing the diseases themselves, across GWAS datasets, to explore their “shared pathogenetics”. The method applies principal component analysis (PCA) to gene-level significance scores across all genes and across GWASs, thereby revealing shared pathogenetics between diseases in an unsupervised fashion. Importantly, it adjusts for potential sources of heterogeneity present between GWAS which can confound investigation of shared disease etiology. We applied *disPCA* to 31 GWASs, including autoimmune diseases, cancers, psychiatric disorders, and neurological disorders. The leading principal components separate these disease classes, as well as inflammatory bowel diseases from other autoimmune diseases. Generally, distinct diseases from the same class tend to be less separated, which is in line with their increased shared etiology. Enrichment analysis of genes contributing to leading principal components revealed pathways that are implicated in the immune system, while also pointing to pathways that have yet to be explored before in this context. Our results point to the potential of *disPCA* in going beyond epidemiological findings of the co-occurrence of distinct diseases, to highlighting novel genes and pathways that unsupervised learning suggest to be key players in the variability across diseases.

## Author Summary

Epidemiological studies have revealed distinct diseases that tend to co-occur in individuals. As genome-wide association studies (GWASs) have increased in numbers, more evidence regarding the genetic nature of this shared disease etiology is revealed. Here, we present a novel method that utilizes principal component analysis (PCA) to explore the relationships and shared pathogenesis between distinct diseases and disease classes. PCA groups and distinguishes between data points by uncovering hidden axes of variation. Applying PCA to 31 GWASs of autoimmune diseases, cancers, psychiatric disorders, neurological disorders, other diseases and body mass index, we report several findings. Diseases of similar classes are located near each other, supporting the genetic component of shared disease etiology. Genes that contributed to distinguishing between diseases are enriched for various pathways including those related to the immune system. These results further our knowledge of the genetic component of shared pathogenesis, highlight possible pathways involved and provide new guidelines for future genetic association studies.

This is a *PLOS Computational Biology* Methods article.

## Introduction

Comorbidity studies show that some distinct diseases tend to co-occur [1]–[6], pointing to a shared genetic and/or environmental component. In the era of genome-wide association studies (GWASs), direct evidence of shared genetic risk factors of diseases comes to light [7]. For example, while it has been previously shown that rheumatoid arthritis and type-1 diabetes co-occur [1], GWASs have identified 12 genes associated with both diseases [8]–[16]. More broadly, disease genes obtained from the Online Mendelian Inheritance in Man [17] were used to assemble the Human Disease Network (HDN) [18], [19], a visual representation of genetic similarity between diseases. Pleiotropy of complex diseases and traits has also been explored by searching genome-wide for variants implicated in more than one disease [16], [20], [21]. Such studies promise to reveal shared genes and offer an expanded understanding from a genetic standpoint of why some diseases tend to co-occur.

Methods for exploring shared genetic risk factors between diseases belong to two main categories (see also recent review [7]). The first category of methods focuses on finding individual variants that are associated with a pair or more of diseases being investigated. In one set of such methods, a GWAS is carried out on a pooled set of individuals with different diseases [10], [16], [20], [21], or by analyzing information for multiple diseases available for the same individuals [22], [23]. Alternatively, and based only on summary statistics of the association test for each single nucleotide polymorphism (SNP), one can simply combine p-values from several GWASs using Fisher's method [24]. The CPMA (cross-phenotype meta-analysis) statistic [25] is another statistic that tests whether a SNP is associated to more than one phenotype. In addition, methods such as the conditional false discovery rate or mixed-models for multiple traits have used known pleiotropy between diseases or traits to increase power [26], [27]. Studies employing these methods have found shared associations between pairs of diseases such as Crohn's disease and celiac disease [16], other autoimmune disease pairs [20], [21], bipolar disorder and schizophrenia [26] and multiple sclerosis and schizophrenia [28]. They have additionally shown that SNPs associated with one autoimmune disease are likely to be associated to other (though not all) autoimmune phenotypes [25].

The second category of methods focuses on using shared variants to learn about the genetic similarity between diseases. One method employed by Sirota *et al.* utilizes the correlation between association signals across many SNPs to assess the similarity between pairs of diseases and showed that there are likely two distinct autoimmune classes where a risk allele for one class may be protective in another [29]. Similar methods based on classifier [30] and linear mixed model approaches [27], [31] have also been proposed for assessing the shared genetic variation between two diseases.

These exciting new methods are powerful for studying shared genetic risk variants between diseases. At the same time, overcoming some of their limitations can improve the study of shared pathogenesis using data from multiple GWASs. First, some methods have focused on analysis of individual SNPs. Though well suited for scenarios of a single causal SNP in a locus, such methods would suffer a reduction in power when several causal SNPs exist or if different SNPs tag the same underlying causal variant, which is especially relevant for diseases with rare causal variants [32], [33] and when the different GWASs are across different populations [34] or have used different genotyping arrays. Second, when considering the correlation between association statistics of different studies, it might be beneficial to not consider all variants equally (as is the case in [29]), whether or not they play a role in disease susceptibility. Third, most methods assume as known which diseases share pathogenesis, and while the shared pathogenesis of autoimmune disease has been well established [25], [29], it is worthwhile to study shared pathogenesis of other disease classes [6], [35], [36]. And fourth, while some approaches perform well for two correlated traits or diseases, extending the analysis to more than two traits can become difficult [27].

In this study, we present a novel method, *disPCA*, which uses principal component analysis (PCA) to learn about the shared genetic risk of distinct diseases. PCA maps data from the original axes into new axes in principal component (PC) space via a stretch and rotation of the original axes. Each new axis or PC captures the maximal level of variation in the data not captured by previous PCs. Thus, each PC can potentially capture a different, orthogonal story told by the data. Our method is based on summary level statistics from GWASs of different diseases. We combine data from individual SNPs into gene-based statistics via several p-value combination methods. PCA is applied to a matrix across genes and GWAS datasets, with entries representing the strength of association between a gene and the disease studied in a dataset. Thus, disPCA reveals principal components that are linear combinations of all genes, weighed in accordance with their role in differentiating between the different GWASs. It can be applied to study multiple diseases without prior knowledge of their shared pathogenesis, thereby overcoming all the limitations of existing methods outlined above. *disPCA* also accounts for potential confounders due to methodological differences between studies, such as in genotyping array, which can otherwise lead to these differences being captured by the PCA.

Equipped with this novel method and with data from 31 GWAS datasets, we considered the level of shared pathogenesis between diseases and classes of diseases from all genes, which we term *shared pathogenetics*. Diseases with more similar underlying genetics are more likely to be located closer together in PC space. As PCA is a non-parametric method, it makes no assumptions regarding which diseases are more similar and does not aim to model it, thereby allowing discovery of new relationships between diseases by examining the top PCs. Each PC captures a different combination of genes that distinguish well between some diseases, or the remaining variation between diseases. No separation between diseases along a PC indicates that they tend to share the pathogenetics underlying that PC. By studying the set of genes underlying each PC for enrichment in specific pathways, we further assessed the function and relationship of genes that separate different disease clusters in PC space.

## Materials and Methods

### *disPCA*

We developed a method, *disPCA*, for studying the relationship between diseases based on their level of disease risk genes shared. The method works on the gene-level by first combining information from all SNPs in and around each gene. Considering gene-level statistics compensates for different tag SNPs being associated in different datasets even in cases where they capture the same causal variant. It also aggregates information across multiple tag SNPs in each dataset, as well as allows for different underlying causal variants in the same gene being associated with the risk of different diseases. To be widely applicable, *disPCA* is based solely on the p-values of association of each SNP with the disease under study. Importantly, all SNPs and consequently all genes are considered, rather than focusing on genes that meet a genome-wide significance level of association with a disease. We apply PCA to many different GWASs to axiomatically find and assign importance to genes based on their contribution to distinguishing between diseases and disease classes. The ensuing distance between different disease datasets in PC space inversely corresponds to their level of shared pathogenetics.

### Gene-level significance levels

For each protein-coding gene from the HGNC database [37], we mapped all SNPs that are in the gene or within 0.01 cM from it (genetic distances were determined via the Oxford genetic map based on HapMap2 data [38], [39]). We discarded all SNPs that were not mapped to within 0.01 cM of any gene. If a SNP lay between two genes, it was assigned to the closer gene. For each GWAS dataset, we determined the significance of association of each gene with the assayed disease using the following simulation procedure. Let the observed p-value of a gene be the minimum p-value of the *n* SNPs mapped to the gene. We compared the observed p-value to that of 100,000 groups of *n* consecutive SNPs chosen in random. Based on these groups, we assign a new p-value to each gene as the proportion of groups for which the observed minimum p-value for that gene is less significant than that of the group. This random sampling procedure may be biased in regions of high linkage disequilibrium (LD) when mapping SNPs to genes using genetic distance (e.g. consecutive SNPs in regions of high LD will be more correlated than those in regions of lower LD). However, for any given gene, these will equally affect each of the datasets. To validate this, we also applied *disPCA* to p-values obtained from mapping SNPs to genes using physical distance: a SNP was mapped to a gene if it was in the gene or within 10 kb of it. Comparing these results to results based on mapping via genetic coordinates revealed the same clustering of diseases (Figure S1). Furthermore, in studying the loading of each gene, namely their contribution to each PC, we found that the genes with the top 50 average loadings on the first two PCs were significantly correlated (r>0.67, p-value<8.4×10^−8^, Table S1). Thus, in the main text we present results based on mapping by genetic distance as described above.

To consider information from beyond only the most significant SNP in a gene, we also implemented the truncated tail strength [40] and the truncated product methods [41] to combine p-values in each gene in replacement of the minimum p-value, and followed a similar procedure for assigning new gene-level p-values. For the analyses presented in the following, results from all methods were similar though results with the minimum p-value approach clusters similar diseases better (Figure S2, S3). We thus only report in the main text results from the minimum p-value approach. Code to carry out this procedure is publicly available at http://keinanlab.cb.bscb.cornell.edu/content/tools-data.

### PCA implementation and confounders

Assume a matrix Z, a *d*×*g* matrix of the −log_10_ gene-level p-values, where *d* is the number of GWAS datasets, and *g* is the number of genes present in all datasets. We center the matrix by subtracting the column means from each column. Thus the centered matrix *B* has entries:
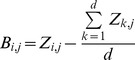
(1)


To obtain the PCs of matrix *B*, we must find the eigenvectors and eigenvalues of its covariance matrix *BB^T^*. Let *v_i_* be a vector of length *d* and let 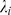 be a scalar. *v_i_* is the eigenvector and *λ* the eigenvalue of *BB* ^T^ if the following is satisfied:
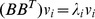
(2)


The principal components of *B* are the normalized eigenvectors of its covariance matrix, *BB^T^*, where the eigenvectors are ordered such that the largest eigenvalue corresponds to the first principal component. Each eigenvector is additionally orthogonal to all other eigenvectors. Thus, from (2), we can decompose *BB^T^* as follows:
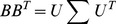
(3)Where the columns of *U* contain the principal components and ∑ is a diagonal matrix with entries equal to the eigenvalues of *B*'s covariance matrix. One can similarly construct the singular value decomposition (SVD) of *B*. The SVD of *B* can be written as:
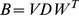
(4)where *V* is a *d*×*d* matrix, *D* is a *d*×*g* diagonal matrix, and *W* is a *g*×*g* matrix. *V* and *W* contain the left and right singular vectors of *B*, respectively, and *D* contains the singular values of *B* in its diagonal. Substituting equation (4) for *B* in equation (3), we find that
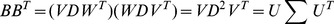
(5)Thus, the principal components of *B*, the eigenvectors of its covariance matrix, are equivalent to the left singular vectors of *B*. In addition, the eigenvalues of *B* are equivalent to the square of its singular values.

We applied SVD to the matrix *B* using the R [42] implementation of PCA/SVD (*prcomp*), with no scaling of the data. Due to the heterogeneity of the GWAS datasets (Table S2), variation uncovered by PCA can also reflect differences in features such as genotyping array, association method, and sample size, rather than underlying disease risk genes. To ensure that these features did not influence our results, we first tested each gene for association with each of these features. Let *z_i_ = Z_i_*_,·_ be the vector corresponding to the association statistic for gene *i* across the *d* datasets. We considered a linear regression of *z_i_* as a function of the covariates: 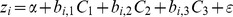, where *C_1_*, *C_2_*, *C_3_* are vectors of length *d* that represent the genotyping array, association method and the log_10_ of the sample size respectively, in each of the studies (Table S2). Testing the significance of regression coefficients can reveal genes that are associated with any of these potential confounders. In our following analysis, 19 genes were significantly associated with association method. However, genes not significantly associated to the above confounders may similarly have an effect. Hence, we also applied SVD (as described above) to the residualized matrix, namely matrix *R* with rows 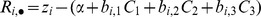. We found that applying SVD to *R* results in the top PCs capturing a higher fraction of the variance of the data than when applied to the original matrix *Z*, though results are qualitatively similar between the two. We thus present results derived from the residualized matrix *R*. Resulting distances between datasets were assessed visually by plotting datasets in PC space. To quantify the clustering of datasets, we additionally applied hierarchical clustering in R [42] (*hclust*) to the Euclidean distance between pairs of datasets across the first two PCs.

### Simulation study

We simulated a matrix *Z* for two disease classes, each with 5 diseases (*A_1_,A_2_,A_3_,A_4_,A_5_,B_1_,B_2_,B_3_,B_4_,B_5_*) and 10,000 genes. In general, under the null hypothesis of a region containing no risk variant and assuming no confounding factors (e.g. population stratification), p-values should be uniformly distributed between 0 and 1. On the other hand, associated risk variants should be enriched for smaller p-values. We thus considered three sets of genes. The p-values for the first set of genes was drawn from the *U*(0,1) distribution for all diseases, thus no pleiotropy was captured in this set of genes. The second set of genes was distributed *U*(0,0.05) for the first disease class (*A_1_,…,A_5_*) and distributed *U*(0,1) for the second disease class (*B_1_,…,B_5_*). Finally the third set of genes was distributed *U*(0,0.05) for the following diseases: *A_1_*, *A_2_*, *B_1_*, *B_2_* and distributed *U*(0,1) for all other diseases. Thus the second set of genes simulates pleiotropy between diseases in disease class A, while the last set of genes simulates pleiotropy between diseases in both disease classes.

### Disease and pathway enrichment analysis

Disease enrichment analysis was completed using the online tool WebGestalt [43], [44] to query the PharmGKB [45] database. WebGestalt tests for enrichment of a category of genes in the observed set of genes using the hypergeometric test [43]. Bonferroni correction for multiple tests was applied and all reported p-values are following this correction. We restricted analysis to categories that contained a minimum of 5 genes in our analysis with the largest 50 weightings in the top two PCs. For gene categories with overlapping or the same set of genes, we list the most significant category. To reduce biases introduced by the clustering of genes with similar function, we filtered our list of genes with the top 50 loadings on the top two PCs by removing the latter gene out of a pair of genes within 0.1 cM of each other. We then applied WebGestalt to this filtered subset of genes.

Pathway enrichment analysis was completed using the Gene Set Enrichment Analysis (GSEA) tool [46]. GSEA sorts genes according to a score, which here is the weighting of a gene in the PC under study. It then assesses whether genes belonging to a certain category (e.g. pathway) are non-randomly distributed in the sorted list. As input to GSEA, we utilized the weights of genes in the top two PCs. GSEA carried out 10,000 gene-set permutations to determine FDR (false discovery rate) q-values. We queried the BioCarta and KEGG pathway databases. We restricted analysis to categories that contained a minimum of 5 genes in our analysis. Throughout we present enrichment analysis only for the top two PCs, though other PCs are available and can be assayed for further insight into the diseases studied. We considered an FDR of 0.25, suggested by GSEA [46] (GSEA manual online), though this entails that 1 in 4 of our results are false positives on average. As above, to reduce biases introduced by the clustering of genes with similar function, we filtered our full list of genes by removing the latter gene out of a pair of genes within 0.1 cM of each other and reanalyzed this subset of genes (n = 5,298) with GSEA.

### Testing for non-random distribution of p-values

We followed a similar approach to that implemented in Zhernakova *et al*. 2011 [21] while applying it to genes instead of individual SNPs to test for non-random distribution of association values. For each disease pair we retained all *k* genes that were nominally significant in one disease (p-value<0.01). We then tested the null hypothesis of a uniform distribution of p-values in the second disease using Fisher's method for combining p-values: 
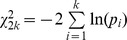
, where *p_i_* is the p-value for association of gene *i* in the second disease. Nearby genes in linkage disequilibrium may violate the independency assumption in Fisher's method. We thus performed a separate analysis after removing the latter of the two genes that were within 0.1 cM of each other and nominally significant in one disease.

### Application of *disPCA* to 31 GWAS datasets

We analyzed a total of 31 GWAS datasets [10], [47]–[76] that spanned different types of cancers, autoimmune diseases, neurological disorders, psychiatric disorders, type-2 diabetes (T2D), ischemic stroke and body mass index (BMI) (Table S2). Datasets were publicly available, obtained from dbGaP or obtained via collaborations. These datasets had non-overlapping samples and were of European ancestry only. For Wellcome Trust Case Control (WT) related datasets, we distributed controls between the five datasets such that none had overlapping samples. For WT type-1 diabetes, rheumatoid arthritis and Crohn's disease, we obtained further controls from the WT hypertension, cardiovascular disease and bipolar disorder case data [10]. After obtaining gene-level association statistics for 14,018–17,438 autosomal genes for each dataset, we limited our analysis to the 11,927 genes that overlapped all studies. Nineteen of these genes were significantly associated with association method after multiple-testing correction (see above).

### Replication of *disPCA*

We tested the replicability of *disPCA* when applied to real GWASs using six datasets for which we had access to the original data [10], [57], [60], [61], [74], [75]. Each dataset was split into independent subsets of equal size (+/− two samples). We then used PLINK's logistic regression [77] to evaluate association of each SNP to disease risk. We additionally incorporated covariates derived from EIGENSOFT into the regression analysis [78] to control for population structure. We randomly chose one subset of each of the six datasets for one *disPCA* analysis, and the rest for another. Hence, these two analyses consist of independent samples.

## Results

**Figure 1:**
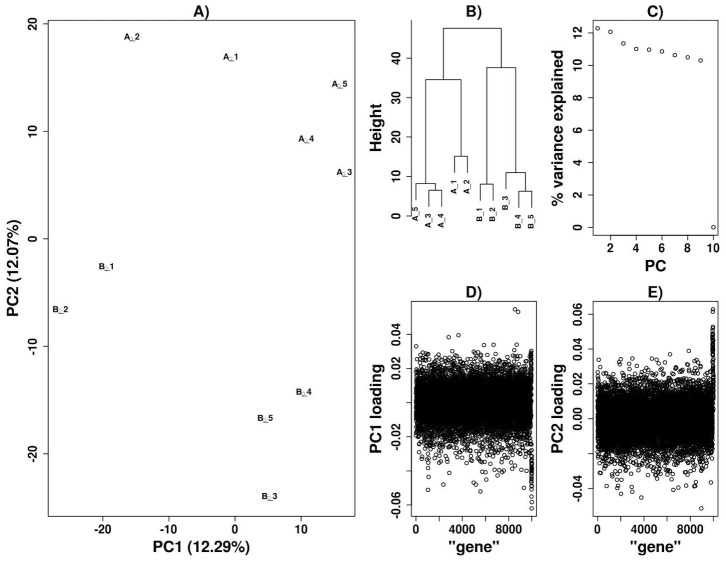
*disPCA* of ten simulated diseases. The p-values for ten diseases were simulated for 10,000 genes (Materials and Methods). Class *A* diseases had p-values uniformly distributed between 0 and 0.05 for 40 of the 10,000 genes, while two diseases from class *A* (*A_1, A_2*) and two diseases from class *B* (*B_1, B_2*) had p-values similarly distributed for a separate set of 40 genes (Materials and Methods). All other genes had p-values that were randomly distributed in each disease between 0 and 1. A) The simulated data is displayed on PC1 and PC2. PC1 separates (*A_1, A_2, B_1, B_2*) from all other diseases, while PC2 separates class *A* diseases from class *B* diseases. B) Dendrogram derived from a clustering analysis based on the Euclidean distance between datasets in the space of the first two PCs (represented as the height of the branches). C) PC1 and PC2 account for a similar amount of variance. D) Loadings for each gene are displayed sequentially for PC1. The 40 genes contributing to pleiotropy between the two diseases in each class (displayed as the last 40 genes) are enriched for larger absolute loadings. E) Similar to (D), with loadings for PC2 displayed. A separate set of 40 genes contributing to correlation between diseases in each class are also enriched for larger loadings.

We first applied *disPCA* to a simulated dataset (Materials and Methods). We varied the number of genes that have correlated association results across simulated datasets, thereby varying the level of pleiotropy between the simulated diseases. *disPCA* clearly clustered pleiotropic diseases when diseases shared at least 40 shared genes with p-values randomly distributed below 0.05 in each disease (Figure 1a–b, S4, S5, S6). This can be seen both visually via PCA plots, and via hierarchical clustering based on the Euclidean distance between datasets in the presented space of the first two principal components (PCs) (Figure 1, S4, S5, S6). When diseases are indeed clustered by their simulated pleiotropy according to *disPCA* (Figure 1b), the first two PCs explain a similar fraction of the variance (Figure 1c), which may increase or decrease depending on the number of genes contributing to pleiotropy (Figure S7). We next examined the contribution of each gene to each PC as captured by its absolute “loading”. Considering the first two PCs in this *disPCA* analysis, genes with p-values<0.05 (Materials and Methods) are also enriched for larger absolute loadings, stressing their role in differentiating between the simulated disease classes (Figure 1d–e).

**Figure 2:**
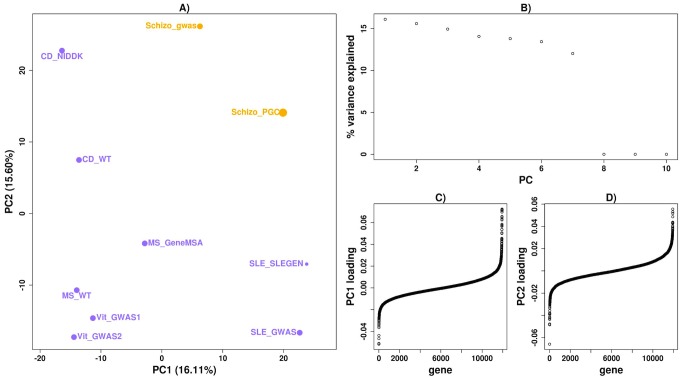
*disPCA* of datasets of the same disease. A) Pairs of datasets of the same autoimmune diseases and schizophrenia are displayed on PC1 and PC2. Dataset labels are indicated in the form of *disease-type*_ *study-name*. The size of points is proportional to the sample size of the original study (Table S2). Diseases include systemic lupus erythematosus (SLE), vitiligo (Vit), multiple sclerosis (MS), schizophrenia (Schizo) and Crohn's disease (CD). Datasets of the same diseases tend to lie closer together on PC1 and PC2. B) The portion of variance explained by each PC is displayed. C) The weightings for genes on PC1 are displayed and ordered according to their weights. D) Similar to (C) with loadings for PC2.

**Figure 3:**
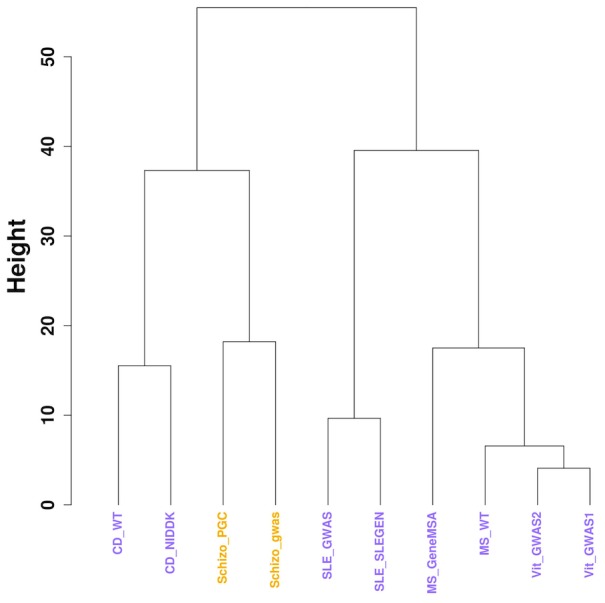
Clustering dendrogram of datasets of the same disease. Each pair of datasets of the same disease cluster together based on hierarchical clustering applied to the Euclidean distance between datasets in the first two PCs presented in Figure 2 (Materials and Methods). The height of the branches represents the Euclidean distance between datasets in the space of the first two PCs.

We next applied *disPCA* to empirical data from GWAS datasets. First, we considered only diseases for which we had two datasets: autoimmune diseases (for which we had the most pairs of datasets) and a pair of schizophrenia datasets (as schizophrenia has a high heritability [79]). We observed that datasets of the same diseases were generally clustered together (Figure 2–3). We additionally observed that Crohn's disease is separated from other autoimmune diseases. This result is consistent with previous reports that inflammatory bowel disorders (IBDs) are distinct from other autoimmune disorders [29]. As in the simulated scenarios, the variance explained by each PC was similar (Figure 2b), and the results suggest that less than a hundred genes contribute to the similarity between each pair of datasets (Figure 3c–d).

To test the replicability of the results, we further divided each of the six datasets, for which we had the raw data, into two subsets consisting of the same or similar number of cases and controls (Materials and Methods). We then performed two *disPCA* analyses, one based on a randomly chosen subset of each of the six datasets, and another based on the remaining subset of each dataset. We found that both independent sets produced the same clustering of diseases (Figure S8, S9). Loadings for 50 genes with the largest average loading across the two *disPCA* analyses of PC1 and PC2 were also significantly correlated across the two (r>0.44, p-value<1.2×10^−3^, Table S3). These results point to *disPCA* capturing some of the same pleiotropy in both cases, and further support the replicability of its results.

**Figure 4:**
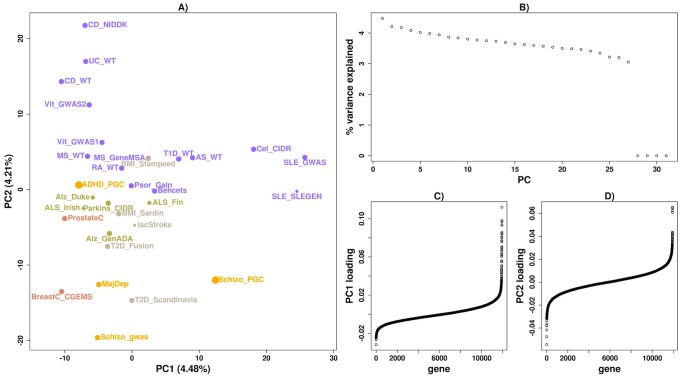
*disPCA* of all diseases and traits. A) Autoimmune diseases (purple), cancers (pink), psychiatric disorders (yellow), neurological disorders (green), and other diseases and traits (grey) are shown on PC1 and PC2. PC1 accounts for 4.48% of the variance, while PC2 accounts for 4.21%. Additional diseases include Alzheimer's disease (Alz), amyotrophic lateral sclerosis (ALS), ankyolosing spondylitis (AS), attention deficit hyperactivity disorder (ADHD), Behcet's disease (Behcets), body mass index (BMI), breast cancer (BreastC), celiac disease (CeliacD), ischemic stroke (IscStroke), major depression (MajDep), Parkinson's disease (Parkin), prostate cancer (ProstateC), psoriasis (Psor), rheumatoid arthritis (RA), type-1 diabetes (T1D), type-2 diabetes (T2D), ulcerative colitis (UC). PC1 clusters celiac disease and SLE together, while PC2 separates inflammatory bowel diseases from other diseases and traits. B) The portion of variance explained by each PC is displayed. Three additional PCs explain 0% of the variance corresponding to the number of confounders we accounted for (Materials and Methods). C) The weightings for genes on PC1 are displayed and ordered according to their weights. D) Similar to (C) where loadings are for PC2.

**Figure 5:**
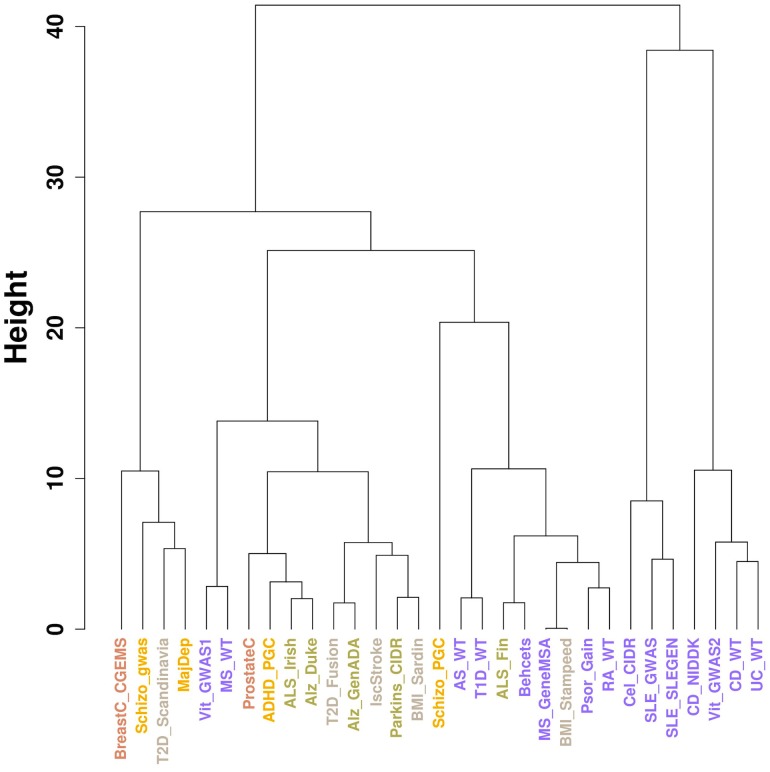
Clustering dendrogram of datasets of all diseases and traits. Dendrogram derived from hierarchical clustering analysis applied to distance (in PC space) between datasets presented in Figure 4. Inflammatory bowel diseases are clustered together, in addition to SLE and celiac disease.

We applied *disPCA* to a final set of 31 datasets (Table S2), including autoimmune diseases, cancers, obesity-related diseases and traits, psychiatric disorders and neurological disorders. The first two PCs capture visually-interpretable separation of diseases. PC1 for the most part splits the two systemic lupus erythematosus (SLE) and the one dataset of celiac disease from all other datasets (Figure 4). Independent of that separation, PC2 splits autoimmune diseases (in purple) from other diseases, and within autoimmune diseases, inflammatory bowel disorders (Crohn's disease and ulcerative colitis) are clustered together (Figures 4–5). Schizophrenia, major depressive disorder, cancers, T2D and neurological disorders lie on the negative end of PC2, while attention deficit hyperactivity disorder (ADHD), and some autoimmune diseases that are not well separated on this PC from other diseases, lie near the origin. PCs beyond the first two explain almost the same fraction of the variance (Figure 4b) and hence merit further investigation (see Discussion).

As *disPCA* teases out the important genes of shared and distinct pathogenetics across disease datasets, we next investigated which genes strongly contribute to each PC based on their absolute loadings. Specifically, we retrieved the genes with the top 50 absolute loadings for each of the top two PCs underlying Figure 4 and tested their disease enrichment (Materials and Methods). The top genes underlying the first PC were significantly enriched for genes associated with lupus and autoimmune related diseases, while genes underlying the second PC were mostly enriched for association to IBD (Table 1). These enrichment results are consistent with the separation of studies across each of these 2 PCs with PC1 mostly separating studies of SLE and celiac diseases, and PC2 mostly separating studies of IBD from other diseases. The results were largely unchanged following filtering genes that were within 0.1 cM of each other to account for linkage disequilibrium and for similar genes being co-located to each other, such as gene families (Table 1) (Materials and Methods).

**Table 1:** Disease enrichment analysis for *disPCA* (Figure 1).

PC	Disease	P-value*	P-value (distance pruned)*
**1**	Lupus erythematosus	1.59×10^−6^	3.0×10^−8^
	Arthritis	1.72×10^−6^	>0.01
	Connective tissue diseases	5.00×10^−4^	>0.01
	Autoimmune diseases	2.6×10^−3^	2.05×10^−6^
	Rheumatic Diseases	2.6×10^−3^	>0.01
	Immune system diseases	6.5×10^−3^	2.2×10^−5^
**2**	Gastroenteritis	5.79×10^−13^	2.92×10^−9^
	Crohn's Disease	2.12×10^−12^	1.73×10^−8^
	Inflammatory bowel diseases	1.65×10^−11^	7.53×10^−8^
	Fistula	4.00×10^−9^	1.37×10^−7^
	Gastrointestinal diseases	3.49×10^−8^	7.16×10^−8^
	Celiac disease	2.75×10^−5^	7.8×10^−6^
	Multiple sclerosis	2×10^−3^	7×10^−4^
	Skin diseases, genetic	2.3×10^−3^	8.1×10^−3^
	Rheumatic diseases	6.4×10^−3^	2.3×10^−3^
	Autoimune diseases	9.6×10^−3^	2.7×10^−3^

*Bonferroni adjusted for multiple testing.

Table shows disease enrichment results for all diseases significantly enriched with an adjusted p-value<0.01. The distance pruned p-values refers to disease enrichment after removing the latter out of a pair of genes that were within 0.1 cM of each other.

Though the results of the disease enrichment analysis support that *disPCA* extracts biologically relevant signals, the arbitrary cutoff of the 50 top genes goes against the potential of PCs being linear combinations of all genes. We thus used GSEA [46], which supports analyzing a pre-ranked list of all genes, to perform pathway enrichment of each PC. GSEA assesses whether genes belonging to a certain pathway are non-randomly distributed in the list of pre-ranked genes. We ranked all genes by the absolute loading in the PC under study. Results of this pathway analysis revealed enrichment for immune related pathways on the first 2 PCs (Table 2) at an FDR of 0.25. The top two pathways enriched on PC1 were the antigen processing and presentation and the intestinal immune network IgA production pathways, which are crucial immune-related pathways. In particular, intestinal IgA antibodies may have a role in celiac disease [80] and inflammatory bowel disease [81], [82]. On PC2, the most significant pathway was the NOD-like receptor signaling pathway. NOD-like receptors have been associated to Crohn's disease, while other immune-related genes likely interacting with NOD2 have been associated to ulcerative colitis [83]. Other immune system pathways were enriched, including the Fc epsilon RI signaling pathway that is related to the antibody IgE, which induces inflammatory response [84]. Two enriched pathways are related to neurons (i.e. the neurotrophin signaling pathway and the Trk-A pathway). In particular, the neurotrophic factor *BDNF* (brain-derived neurotrophic factor), which is a part of the neurotrophin pathway, has been previously associated to Alzheimer's, Parkinson's disease and depression [85]–[87]. More recently, an intronic variant in this gene has also been associated to BMI [88]. The contribution of genes in these pathways to PC2 may explain the separation of neurological, psychiatric and BMI studies along that PC. As above, we reran GSEA after filtering genes that were within 0.1 cM of each other (Materials and Methods). The top two pathways on the first PC remained significant, while only the top pathway in PC2 remained significant (Table S4). This is likely due to the contribution to enrichment of several genes that are co-located, which should hence not necessarily be discounted.

**Table 2:** Gene enrichment analysis for *disPCA*.

PC	Pathway	FDR (q-value)
**1**	Antigen processing and presentation	0.034
	Intestinal immune network for IgA production	0.042
	Trk-A pathway	0.169
	CK1 pathway	0.213
	DREAM pathway	0.228
	Valine leucine and isoleucine biosynthesis	0.228
	O-glycan biosynthesis	0.243
	Folate biosynthesis	0.246
**2**	NOD-like receptor signaling pathway	<1×10^−4^
	Intestinal immune network for IgA production	0.074
	Neurotrophin signaling pathway	0.165
	Chemokine signaling pathway	0.195
	Fc epsilon RI signaling pathway	0.232
	Terpenoid backbone biosynthesis	0.232
	JAK-STAT signaling pathway	0.238

Table shows pathways that are enriched in the *disPCA* analysis based on the GSEA analysis.

**Figure 6:**
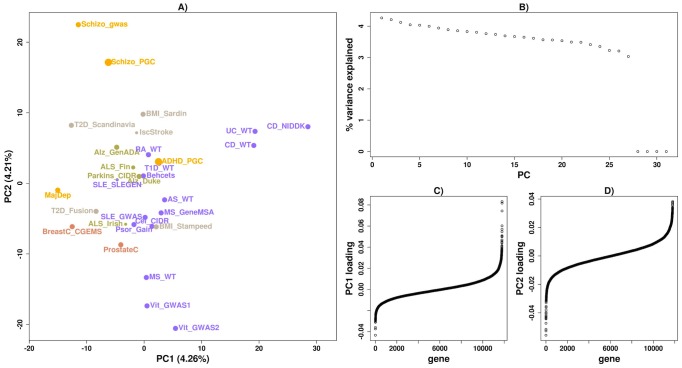
*disPCA* of all diseases and traits excluding the HLA and surrounding region. A) Similar to Figure 4 where genes in the HLA and surrounding region were removed. Though IBD remains separated as in the original *disPCA*, the clustering of celiac disease and SLE is no longer captured by the top two PC's. B) The portion of variance explained by each PC is displayed. C) The weightings for genes on PC1 are displayed and ordered according to their weights. D) Similar to (C) where loadings are for PC2.

Many autoimmune diseases share associations from the HLA region. We thus reran *disPCA* after removing all genes in and around the HLA region, and found a slightly different visual PCA map (Figure 6). SLE and celiac disease were no longer distinguished from other autoimmune diseases and instead lay near the origin. PC1 now differentiated IBD from other diseases, and PC2 separated some autoimmune diseases from the rest on one extreme, and schizophrenia from the rest on the other. This was further supported by clustering results on the first two PCs (Figure S10). A GSEA analysis of the PC loadings retained the NOD-like receptor signaling pathway on PC1 instead of PC2 (Table 3). Analysis of PC2 loadings revealed additional immune related pathways that were not enriched in our previous analysis that included the HLA region.

**Table 3:** Gene enrichment analysis for *disPCA* without the HLA region.

PC	Pathway	FDR q-value
**1**	NOD-like receptor signaling pathway	0.006
	Local acute inflammatory response pathway	0.143
**2**	Proteasome pathway	0.077
	Th1–Th2 pathway	0.102
	Proximal tubule bicarbonate reclamation	0.135
	Adherens junction	0.142
	RNA polymerase	0.171
	CTLA-4 pathway	0.173

Table shows pathways that are enriched in the *disPCA* analysis based on the GSEA analysis after removing genes in the HLA and surrounding region.

**Figure 7:**
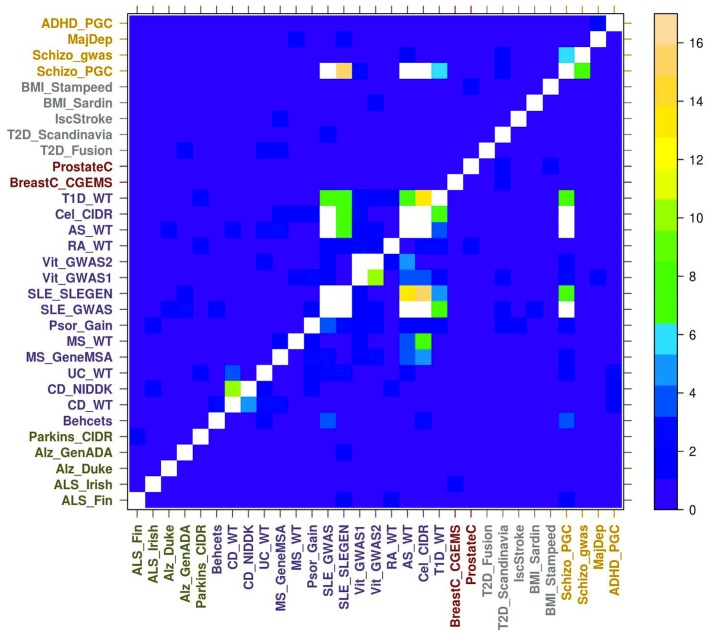
Non-random distribution of genes for all analyzed datasets from Figure 4. Genes nominally significant for diseases on the *y*-axis were tested for non-random distribution in diseases on the *x*-axis (Materials and Methods), with −log_10_ presented on the color scale on the right. White entries denote p-values<1×10^−17^. The most significant results are for pairs of similar diseases and between pairs of autoimmune diseases. In addition, pairs between some autoimmune diseases and schizophrenia also display significant results.

Results such as PC1 in the main analysis clustering schizophrenia close to some autoimmune diseases (Figure 4) prompted us to further explore the shared pathogenetics between diseases by testing for the non-random distribution of gene-based p-values in one disease based on their nominal significance in another disease (Materials and Methods). Generally, the results show that association statistics are non-randomly distributed when considering most pairs of autoimmune diseases, i.e. testing for non-random distribution in one autoimmune disease dataset based on significance in another autoimmune disease dataset (Figure 7). As a control, we tested for non-random distribution for a random set of genes and found that no disease pair was significant for non-random distribution (Figure S11). Our results reported a similar story as observed via *disPCA*. Genes nominally significant in rheumatoid arthritis, type-1 diabetes and ankyolosing spondylitis were non-randomly distributed in SLE and vice versa. We also found that genes nominally significant for one schizophrenia study were non-randomly distributed in a number of autoimmune diseases (Figure 7). These signals remained even after genes within 0.1 cM of another gene were removed (Figure S12) (Materials and Methods).

## Discussion

In this study we introduced a new method, *disPCA*, to explore the shared pathogenetics of various diseases and disease classes based on GWAS data. PCA has been widely used in population and medical genetics. Applied to genome-wide genotyping data, it can recapitulate population structure such as revealing European geography [89], has been used as a tool to assess and correct for population stratification in GWAS [78], [90] and has also been proposed as a tool for reducing the dimensionality of multiple phenotypes for association analysis [91]. Our *disPCA* method considers PCA on a different type of matrix, whereby different GWASs are studied in the space of all genes. It can group GWASs of different diseases together based on gene-level association statistics, while accounting for biases due to heterogeneity in sample size, association method, genotyping array and other confounders between studies. This implementation of PCA assigns weights to each gene and each PC in a manner that maximizes the variation between diseases. Hence, the higher the level of shared pathogenetics between diseases, the closer they will be in PC space. This is in contrast to methods that considered the correlation between diseases across all SNPs [29]. In fact, when we consider such correlations in our data, it is generally very low, even when considering it on the gene rather than on the SNP-level and even when the same disease is studied. For example, the correlation coefficient between the −log10 p-values of the two Crohn's disease studies is 0.048, and it is 0.063 and 0.031 between ulcerative colitis and each of the two Crohn's disease studies. More generally, the highest correlation between pairs of datasets of the same disease was obtained for schizophrenia (0.13, p-value = 2.2×10^−16^) while the lowest was obtained for type-2 diabetes (0.0031, p-value = 0.73). These results show that there is less power when aggregating information across all genes and that *disPCA* is able to tease out and weigh the suitable set of genes underlying shared pathogenetics.

Though *disPCA* is designed to uncover shared disease etiology between diseases, other sources of correlation between datasets can also contribute to its results. Potential confounders include shared samples between datasets, technical artifacts, and population structure (if risk factors vary across ancestry). We accounted for technical artifacts introduced by the genotyping array, association method and sample size by regressing out variation in the data attributed to these sources (Materials and Methods). To minimize the impact of population structure and shared samples, we only applied *disPCA* to studies of individuals of European ancestry and datasets that had no overlapping case or control data. Though we cannot account for other potential confounders that are unknown, our results strongly suggest that the remaining correlation between studies represent shared disease etiology.

We applied *disPCA* to data from 31 GWASs that cover a range of diseases in four main classes: autoimmune diseases, cancers, neurological disorders and psychiatric disorders. We additionally analyzed GWASs on T2D, BMI and ischemic stroke. We first observed that different studies of the same diseases tend to lie closer together on the lead PCs (Figure 2). This is in support of studies of the same disease replicating many of the same signals of associations when samples are of similar ancestry. We additionally find that *disPCA* positions diseases within the same class closer together (Figure 4). This was especially the case for the major types of IBDs (i.e. Crohn's disease and ulcerative colitis), which clustered close together (Figure 5). This points to distinct etiology shared between IBDs, that is not shared between IBDs and most other autoimmune diseases. Indeed, it has recently been suggested that IBD is at least in part a primary immunodeficiency disorder [92], [93]. Between the different disease classes, the main 2 PCs in *disPCA* found overlap between non-autoimmune diseases and traits, as well as pointed to a potential connection between schizophrenia and some autoimmune diseases.

Using the weightings of genes on each of the leading PCs, we performed disease and pathway enrichment analysis. We found that PC1, which mainly splits some autoimmune disorders from other autoimmune disorders, is significantly enriched for genes associated to immune and autoimmune disorders. PC2, which splits IBD studies from studies of other diseases, is significantly enriched for genes in some inflammatory related pathways and genes associated with IBD. Further results in PC2 highlighted neuron-related pathways that can be in line with evidence that abnormal neurotrophins levels in the brain have been associated to schizophrenia [94], [95]. Excluding the HLA region revealed significant enrichment for genes in other immune-related pathways. Though the specific analysis presented in this paper focused on the top two PCs, further PCs estimated by *disPCA* can be examined. For example, PC4 of *disPCA* on all GWASs distinguishes rheumatoid arthritis from other diseases (Figure S13). Pathway enrichment analysis highlighted the calcineurin pathway (FDR = 0.182), which is involved in T-cell activation. Additionally, though schizophrenia and vitiligo datasets are further apart on the first two PCs, each pair of datasets is clustered closer together on PC3 and PC4. Altogether, these results support the validity of the enrichment analysis based on *disPCA*. The analysis in turn also raises new hypotheses of disease etiology by pointing to additional pathways and enrichment for other diseases that were not previously observed.

Prompted by the results of *disPCA*, we further explored shared pathogenetics by testing for the non-random distribution of association statistics between pairs of disease studies (Figure 7). Autoimmune diseases show non-random distribution of association statistics with one another. Interestingly, genes nominally associated with one of the schizophrenia studies were non-randomly distributed in studies of several autoimmune diseases (i.e. ankyolosing spondylitis, systemic lupus erythematosus, and T1D), in support of the above *disPCA* results. Interestingly, this relationship was only observed for one of the two schizophrenia studies we analyzed, which may be due to a number of factors, including high number of risk factors for schizophrenia, with different ones being associated in different studies. If indeed autoimmune diseases and schizophrenia share disease etiology, then just as one would not include individuals with ulcerative colitis as controls for a Crohn's disease GWAS since they both are IBDs, one should also be wary of including individuals with autoimmune disorders in a schizophrenia GWAS (and vice versa) as doing so may decrease power in loci implicated in both diseases. Lack of power due to such or other reasons might also underlie our lack of observation of significant shared etiology between the second schizophrenia dataset and autoimmune diseases.

Finally, we make a few recommendations for future applications of *disPCA* to additional studies: (1) Biases can be introduced when studies share sample data; (2) As *disPCA* maximizes variance across diseases, genes that are implicated in all analyzed diseases will not contribute to the lead PC as they do not distinguish diseases from each other; (3) While here we only focused on using the strength of association and on gene-level signals, the method itself is highly flexible. One can further utilize the direction of association (protective versus deleterious), the heritability at each locus [96], an analysis at the pathway-level or in linkage disequilibrium blocks, include other non-genic functional elements, and/or environmental risk factors; (4) *disPCA* can be used to generate new hypotheses, which can then be tested by conducting more focused association studies in independent data or by using its output to better combine different diseases in an independent meta-analysis. New hypotheses can also be generated with regard to the genes that contribute to comorbidity between diseases. In conclusion, *disPCA* offers users a unique general overview of the disease landscape by studying their distinct and shared pathogenetics and flagging pathways and genes for further investigation. *disPCA*'s flexibility and computational efficiency proves itself as an excellent tool to be applied to additional diseases and disease classes to further our knowledge of shared pathogenetics.

## Supporting Information

Figure S1 **Clustering dendrogram of datasets of the same diseases using physical distance mapping.** SNPs were mapped to genes if they were within 10 kb of the gene. Clustering analysis of resulting *disPCA* revealed the same clusters as *disPCA* with genetic coordinates (Figure 3).
(TIFF)

Figure S2 **Clustering dendrogram of datasets of the same diseases with the truncated product method.** Similar to Figure 3, with the truncated product method used to combine SNP p-values per gene.
(TIFF)

Figure S3 **Clustering dendrogram of datasets of the same diseases with truncated tail strength method.** Similar to Figure 3, with the truncated tail strength method used to combine SNP p-values per gene.
(TIFF)

Figure S4 **Simulated diseases with ten nominally significant genes.** A) Similar to Figure 1 in main text with only ten nominally significant genes for each set of pleiotropic diseases (Materials and Methods). Clustering of the diseases sets is not observed. B) Clustering dendrogram as similarly presented in Figure 1b. C) The portion of variance explained by each PC is displayed. D–E) The loadings for PC1 and PC2 are displayed.
(TIFF)

Figure S5 **Simulated diseases with twenty nominally significant genes.** A) Similar to Figure 1 with twenty nominally significant genes for each set of pleiotropic diseases. As in Figure S2, diseases are not clearly clustering according to the sets though nominally significant genes are enriched for larger absolute loadings (Materials and Methods). B) Clustering dendrogram as similarly presented in Figure 1b. C) The portion of variance explained by each PC is displayed. D–E) The loadings for PC1 and PC2 are displayed.
(TIFF)

Figure S6 **Simulated diseases with thirty nominally significant genes.** A) Similar to Figure 1 with thirty nominally significant genes for each set of pleiotropic diseases. The proper clustering of diseases is beginning to emerge. B) Clustering dendrogram as similarly presented in Figure 1b. C) The portion of variance explained by each PC is displayed. D–E) The loadings for PC1 and PC2 are displayed. Genes with nominally significant p-values are enriched for larger absolute loadings.
(TIFF)

Figure S7 **Simulated diseases with 100 and 200 nominally significant genes.** A) Similar to Figure 1 with 100 and 200 nominally significant genes for the two sets of pleiotropic diseases. Disease sets are tightly clustered and the first two PCs explain a larger portion of the variance compared to other PCs. B) Clustering dendrogram as similarly presented in Figure 1b. C) The portion of variance explained by each PC is displayed. D–E) The loadings for PC1 and PC2 are displayed.
(TIFF)

Figure S8 **Clustering dendrogram of Replication Set 1 datasets.** Clustering of the distance in PC space between datasets in Replication Set 1. Diseases include vitiligo (Vit), multiple sclerosis (MS), schizophrenia (Schizo) and Crohn's disease (CD).
(TIFF)

Figure S9 **Clustering dendrogram of Replication Set 2 datasets.** Similar to Figure S8 with datasets from Replication Set 2.
(TIFF)

Figure S10 **Clustering dendrogram of all diseases and traits excluding the HLA and surrounding regions.** Figure is similar to Figure 5, with clustering analysis of distance between datasets based on the *disPCA* between all diseases and traits presented in Table S2 after removing the HLA and surrounding regions.
(TIFF)

Figure S11 **Non-random distribution of randomly chosen genes.** A random subset of genes were chosen to be tested for non-random distribution in diseases on the x-axis, with −log_10_ presented on the color scale on the right. White entries denote p-values<1×10^−17^.
(TIFF)

Figure S12 **Non-random distribution for distance pruned set of genes.** Genes were filtered such that no two genes were within 0.1 cM of another. The remaining subset of genes was then tested for non-random distribution in diseases on the x-axis. The −log_10_ of the p-value is presented on the color scale and white entries denote p-values<1×10^−17^. Results are largely similar to the original without filtering of nearby genes.
(TIFF)

Figure S13 **PC3 and PC4 of all diseases** ***disPCA*** **.** Similar to Figure 4 with data being presented for PC3 and PC4. A) PC1 accounts for 4.18% of the variance, while PC2 accounts for 4.08%. PC1 clusters schizophrenia and vitiligo datasets together on the two extremes, while PC2 separates rheumatoid arthritis from other diseases and traits. B) The portion of variance explained by each PC is displayed. C) The weightings for genes on PC1 are displayed and ordered according to their weights. D) Similar to (C) where loadings are for PC2.
(TIFF)

Table S1 **Comparison of loadings between** ***disPCA*** **with mapping based on physical or genetic coordinates.** Loadings for the top 50 genes ranked by either a physical or genetic coordinates based *disPCA* were compared. ‘Correlation’ denotes the Pearson's correlation coefficient with its significance denoted in the ‘p-value’ column. Rows denoted by ‘mean(PC1,PC2)’ indicate the correlation between the 50 genes with the largest average loading of PC1 and PC2.
(DOC)

Table S2 **Dataset attributes.** Various attributes of datasets utilized in this study.
(DOC)

Table S3 **Comparison of loadings between Replication Sets 1 and 2.** Loadings for the top 50 genes ranked by either Replication Set 1 or Replication Set 2 were compared. ‘Correlation’ denotes the Pearson's correlation coefficient with its significance denoted in the ‘p-value’ column. Rows denoted by ‘mean(PC1,PC2)’ indicate the correlation between the 50 genes with the largest average loading of PC1 and PC2.
(DOC)

Table S4 **Pathway enrichment after filtering nearby genes.** Pathway enrichment was applied to a subset of genes that were located greater than 0.1 cM from each other.
(DOC)

## References

[1] Somers EC, Thomas SL, Smeeth L, Hall AJ (2009) Are individuals with an autoimmune disease at higher risk of a second autoimmune disorder? Am J Epidemiol 169: 749–755. 19224981 10.1093/aje/kwn408

[2] Marrie RA, Horwitz RI, Cutter G, Tyry T, Vollmer T (2011) Smokers with multiple sclerosis are more likely to report comorbid autoimmune diseases. Neuroepidemiology 36: 85–90. 21282965 10.1159/000323948 PMC3047764

[3] Broadley SA, Deans J, Sawcer SJ, Clayton D, Compston DA (2000) Autoimmune disease in first-degree relatives of patients with multiple sclerosis. A UK survey. Brain : a journal of neurology 123 (Pt 6): 1102–1111. 10.1093/brain/123.6.1102 10825350

[4] Sardu C, Cocco E, Mereu A, Massa R, Cuccu A, et al (2012) Population based study of 12 autoimmune diseases in Sardinia, Italy: prevalence and comorbidity. PLoS One 7: e32487. 22396771 10.1371/journal.pone.0032487 PMC3292563

[5] Sowers JR (1998) Comorbidity of hypertension and diabetes: the fosinopril versus amlodipine cardiovascular events trial (FACET). Am J Cardiol 82: 15R–19R. 10.1016/s0002-9149(98)00751-6 9822138

[6] Zaccara G (2009) Neurological comorbidity and epilepsy: implications for treatment. Acta Neurol Scand 120: 1–15. 19527225 10.1111/j.1600-0404.2008.01146.x

[7] Solovieff N, Cotsapas C, Lee PH, Purcell SM, Smoller JW (2013) Pleiotropy in complex traits: challenges and strategies. Nature reviews Genetics 14: 483–495. 10.1038/nrg3461 PMC4104202 23752797

[8] Hindorff LA, Sethupathy P, Junkins HA, Ramos EM, Mehta JP, et al (2009) Potential etiologic and functional implications of genome-wide association loci for human diseases and traits. Proc Natl Acad Sci U S A 106: 9362–9367. 19474294 10.1073/pnas.0903103106 PMC2687147

[9] Hindorff LA, MacArthur J, J M, Junkins HA, Hall PN, et al. (2013) A Catalog of Published Genome-wide Association Studies. Available: http://www.genome.gov/gwastudies/

[10] The Wellcome Trust Case Control Consortium (2007) Genome-wide association study of 14,000 cases of seven common diseases and 3,000 shared controls. Nature 447: 661–678. 17554300 10.1038/nature05911 PMC2719288

[11] Todd JA, Walker NM, Cooper JD, Smyth DJ, Downes K, et al (2007) Robust associations of four new chromosome regions from genome-wide analyses of type 1 diabetes. Nat Genet 39: 857–864. 17554260 10.1038/ng2068 PMC2492393

[12] Barrett JC, Clayton DG, Concannon P, Akolkar B, Cooper JD, et al (2009) Genome-wide association study and meta-analysis find that over 40 loci affect risk of type 1 diabetes. Nat Genet 41: 703–707. 19430480 10.1038/ng.381 PMC2889014

[13] Hakonarson H, Grant SF, Bradfield JP, Marchand L, Kim CE, et al (2007) A genome-wide association study identifies KIAA0350 as a type 1 diabetes gene. Nature 448: 591–594. 17632545 10.1038/nature06010

[14] Stahl EA, Raychaudhuri S, Remmers EF, Xie G, Eyre S, et al (2010) Genome-wide association study meta-analysis identifies seven new rheumatoid arthritis risk loci. Nat Genet 42: 508–514. 20453842 10.1038/ng.582 PMC4243840

[15] Okada Y, Terao C, Ikari K, Kochi Y, Ohmura K, et al (2012) Meta-analysis identifies nine new loci associated with rheumatoid arthritis in the Japanese population. Nat Genet 44: 511–516. 22446963 10.1038/ng.2231

[16] Festen EA, Goyette P, Green T, Boucher G, Beauchamp C, et al (2011) A meta-analysis of genome-wide association scans identifies IL18RAP, PTPN2, TAGAP, and PUS10 as shared risk loci for Crohn's disease and celiac disease. PLoS Genet 7: e1001283. 21298027 10.1371/journal.pgen.1001283 PMC3029251

[17] Hamosh A, Scott AF, Amberger JS, Bocchini CA, McKusick VA (2005) Online Mendelian Inheritance in Man (OMIM), a knowledgebase of human genes and genetic disorders. Nucleic Acids Res 33: D514–517. 15608251 10.1093/nar/gki033 PMC539987

[18] Darabos C, Desai K, Cowper-Sal·lari R, Giacobini M, Graham BE, et al.. (2013) Evolutionary Computation, Machine Learning and Data Mining in Bioinformatics: Springer Berlin Heidelberg.

[19] Goh KI, Cusick ME, Valle D, Childs B, Vidal M, et al (2007) The human disease network. Proc Natl Acad Sci U S A 104: 8685–8690. 17502601 10.1073/pnas.0701361104 PMC1885563

[20] Ellinghaus D, Ellinghaus E, Nair RP, Stuart PE, Esko T, et al (2012) Combined analysis of genome-wide association studies for Crohn disease and psoriasis identifies seven shared susceptibility loci. Am J Hum Genet 90: 636–647. 22482804 10.1016/j.ajhg.2012.02.020 PMC3322238

[21] Zhernakova A, Stahl EA, Trynka G, Raychaudhuri S, Festen EA, et al (2011) Meta-analysis of genome-wide association studies in celiac disease and rheumatoid arthritis identifies fourteen non-HLA shared loci. PLoS Genet 7: e1002004. 21383967 10.1371/journal.pgen.1002004 PMC3044685

[22] Lee PH, Bergen SE, Perlis RH, Sullivan PF, Sklar P, et al (2011) Modifiers and subtype-specific analyses in whole-genome association studies: a likelihood framework. Hum Hered 72: 10–20. 21849790 10.1159/000327158 PMC7077092

[23] Hartley SW, Monti S, Liu CT, Steinberg MH, Sebastiani P (2012) Bayesian methods for multivariate modeling of pleiotropic SNP associations and genetic risk prediction. Front Genet 3: 176. 22973300 10.3389/fgene.2012.00176 PMC3438684

[24] Fisher RA (1925) Statistical Methods for Research Workers. Edinburgh: Oliver and Boyd.

[25] Cotsapas C, Voight BF, Rossin E, Lage K, Neale BM, et al (2011) Pervasive sharing of genetic effects in autoimmune disease. PLoS Genet 7: e1002254. 21852963 10.1371/journal.pgen.1002254 PMC3154137

[26] Andreassen OA, Thompson WK, Schork AJ, Ripke S, Mattingsdal M, et al (2013) Improved detection of common variants associated with schizophrenia and bipolar disorder using pleiotropy-informed conditional false discovery rate. PLoS Genet 9: e1003455. 23637625 10.1371/journal.pgen.1003455 PMC3636100

[27] Korte A, Vilhjalmsson BJ, Segura V, Platt A, Long Q, et al (2012) A mixed-model approach for genome-wide association studies of correlated traits in structured populations. Nat Genet 44: 1066–1071. 22902788 10.1038/ng.2376 PMC3432668

[28] Andreassen OA, Harbo HF, Wang Y, Thompson WK, Schork AJ, et al.. (2014) Genetic pleiotropy between multiple sclerosis and schizophrenia but not bipolar disorder: differential involvement of immune-related gene loci. Mol Psychiatry [epub ahead of print] 10.1038/mp.2013.195 PMC4356743 24468824

[29] Sirota M, Schaub Ma, Batzoglou S, Robinson WH, Butte AJ (2009) Autoimmune disease classification by inverse association with SNP alleles. PLoS Genet 5: e1000792. 20041220 10.1371/journal.pgen.1000792 PMC2791168

[30] Schaub MA, Kaplow IM, Sirota M, Do CB, Butte AJ, et al (2009) A Classifier-based approach to identify genetic similarities between diseases. Bioinformatics 25: i21–29. 19477990 10.1093/bioinformatics/btp226 PMC2687980

[31] Lee SH, Ripke S, Neale BM, Faraone SV, Purcell SM, et al (2013) Genetic relationship between five psychiatric disorders estimated from genome-wide SNPs. Nat Genet 45: 984–994. 23933821 10.1038/ng.2711 PMC3800159

[32] Wang K, Dickson SP, Stolle CA, Krantz ID, Goldstein DB, et al (2010) Interpretation of association signals and identification of causal variants from genome-wide association studies. Am J Hum Genet 86: 730–742. 20434130 10.1016/j.ajhg.2010.04.003 PMC2869011

[33] Chang D, Keinan A (2012) Predicting signatures of “synthetic associations” and “natural associations”. from empirical pattherns of human genetic variation PLoS Comp Biol 8: e1002600. 10.1371/journal.pcbi.1002600 PMC3390358 22792059

[34] Marigorta UM, Navarro A (2013) High Trans-ethnic Replicability of GWAS Results Implies Common Causal Variants. PLoS Genet 9: e1003566. 23785302 10.1371/journal.pgen.1003566 PMC3681663

[35] Yancik R, Havlik RJ, Wesley MN, Ries L, Long S, et al (1996) Cancer and comorbidity in older patients: a descriptive profile. Ann Epidemiol 6: 399–412. 8915471 10.1016/s1047-2797(96)00063-4

[36] McElroy SL (2004) Diagnosing and treating comorbid (complicated) bipolar disorder. J Clin Psychiatry 65 (Suppl 15) 35–44. 15554795

[37] Gray KA, Daugherty LC, Gordon SM, Seal RL, Wright MW, et al (2013) Genenames.org: the HGNC resources in 2013. Nucleic Acids Res 41: D545–552. 23161694 10.1093/nar/gks1066 PMC3531211

[38] Frazer KA, Ballinger DG, Cox DR, Hinds DA, Stuve LL, et al (2007) A second generation human haplotype map of over 3.1 million SNPs. Nature 449: 851–861. 17943122 10.1038/nature06258 PMC2689609

[39] Myers S, Bottolo L, Freeman C, McVean G, Donnelly P (2005) A fine-scale map of recombination rates and hotspots across the human genome. Science (New York, NY) 310: 321–324. 10.1126/science.1117196 16224025

[40] Jiang B, Zhang X, Zuo Y, Kang G (2011) A powerful truncated tail strength method for testing multiple null hypotheses in one dataset. J Theor Biol 277: 67–73. 21295595 10.1016/j.jtbi.2011.01.029

[41] Zaykin DV, Zhivotovsky LA, Westfall PH, Weir BS (2002) Truncated product method for combining P-values. Genet Epidemiol 22: 170–185. 11788962 10.1002/gepi.0042

[42] R Core Team (2013) R: A Language and Environment for Statistical Computing.

[43] Zhang B, Kirov S, Snoddy J (2005) WebGestalt: an integrated system for exploring gene sets in various biological contexts. Nucleic Acids Res 33: W741–748. 15980575 10.1093/nar/gki475 PMC1160236

[44] Wang J, Duncan D, Shi Z, Zhang B (2013) WEB-based GEne SeT AnaLysis Toolkit (WebGestalt): update 2013. Nucleic Acids Res 41: W77–83.. 23703215 10.1093/nar/gkt439 PMC3692109

[45] Whirl-Carrillo M, McDonagh EM, Hebert JM, Gong L, Sangkuhl K, et al (2012) Pharmacogenomics knowledge for personalized medicine. Clin Pharmacol Ther 92: 414–417. 22992668 10.1038/clpt.2012.96 PMC3660037

[46] Subramanian A, Tamayo P, Mootha VK, Mukherjee S, Ebert BL, et al (2005) Gene set enrichment analysis: a knowledge-based approach for interpreting genome-wide expression profiles. Proc Natl Acad Sci U S A 102: 15545–15550. 16199517 10.1073/pnas.0506580102 PMC1239896

[47] Laaksovirta H, Peuralinna T, Schymick JC, Scholz SW, Lai SL, et al (2010) Chromosome 9p21 in amyotrophic lateral sclerosis in Finland: a genome-wide association study. Lancet Neurol 9: 978–985. 20801718 10.1016/S1474-4422(10)70184-8 PMC2965392

[48] Cronin S, Berger S, Ding J, Schymick JC, Washecka N, et al (2008) A genome-wide association study of sporadic ALS in a homogenous Irish population. Hum Mol Genet 17: 768–774. 18057069 10.1093/hmg/ddm361

[49] Heinzen EL, Need AC, Hayden KM, Chiba-Falek O, Roses AD, et al (2010) Genome-wide scan of copy number variation in late-onset Alzheimer's disease. J Alzheimers Dis 19: 69–77. 20061627 10.3233/JAD-2010-1212 PMC2883723

[50] Li H, Wetten S, Li L, St Jean PL, Upmanyu R, et al (2008) Candidate single-nucleotide polymorphisms from a genomewide association study of Alzheimer disease. Arch Neurol 65: 45–53. 17998437 10.1001/archneurol.2007.3

[51] Evans DM, Spencer CC, Pointon JJ, Su Z, Harvey D, et al (2011) Interaction between ERAP1 and HLA-B27 in ankylosing spondylitis implicates peptide handling in the mechanism for HLA-B27 in disease susceptibility. Nat Genet 43: 761–767. 21743469 10.1038/ng.873 PMC3640413

[52] Neale BM, Medland SE, Ripke S, Asherson P, Franke B, et al (2010) Meta-analysis of genome-wide association studies of attention-deficit/hyperactivity disorder. J Am Acad Child Adolesc Psychiatry 49: 884–897. 20732625 10.1016/j.jaac.2010.06.008 PMC2928252

[53] Remmers EF, Cosan F, Kirino Y, Ombrello MJ, Abaci N, et al (2010) Genome-wide association study identifies variants in the MHC class I, IL10, and IL23R-IL12RB2 regions associated with Behcet's disease. Nat Genet 42: 698–702. 20622878 10.1038/ng.625 PMC2923807

[54] Sabatti C, Service SK, Hartikainen AL, Pouta A, Ripatti S, et al (2009) Genome-wide association analysis of metabolic traits in a birth cohort from a founder population. Nat Genet 41: 35–46. 19060910 10.1038/ng.271 PMC2687077

[55] Scuteri A, Sanna S, Chen WM, Uda M, Albai G, et al (2007) Genome-wide association scan shows genetic variants in the FTO gene are associated with obesity-related traits. PLoS Genet 3: e115. 17658951 10.1371/journal.pgen.0030115 PMC1934391

[56] Hunter DJ, Kraft P, Jacobs KB, Cox DG, Yeager M, et al (2007) A genome-wide association study identifies alleles in FGFR2 associated with risk of sporadic postmenopausal breast cancer. Nat Genet 39: 870–874. 17529973 10.1038/ng2075 PMC3493132

[57] Duerr RH, Taylor KD, Brant SR, Rioux JD, Silverberg MS, et al (2006) A genome-wide association study identifies IL23R as an inflammatory bowel disease gene. Science 314: 1461–1463. 17068223 10.1126/science.1135245 PMC4410764

[58] Matarin M, Brown WM, Scholz S, Simon-Sanchez J, Fung HC, et al (2007) A genome-wide genotyping study in patients with ischaemic stroke: initial analysis and data release. Lancet Neurol 6: 414–420. 17434096 10.1016/S1474-4422(07)70081-9 PMC2613843

[59] Boomsma DI, Willemsen G, Sullivan PF, Heutink P, Meijer P, et al (2008) Genome-wide association of major depression: description of samples for the GAIN Major Depressive Disorder Study: NTR and NESDA biobank projects. Eur J Hum Genet 16: 335–342. 18197199 10.1038/sj.ejhg.5201979

[60] Sawcer S, Hellenthal G, Pirinen M, Spencer CC, Patsopoulos NA, et al (2011) Genetic risk and a primary role for cell-mediated immune mechanisms in multiple sclerosis. Nature 476: 214–219. 21833088 10.1038/nature10251 PMC3182531

[61] Baranzini SE, Wang J, Gibson RA, Galwey N, Naegelin Y, et al (2009) Genome-wide association analysis of susceptibility and clinical phenotype in multiple sclerosis. Hum Mol Genet 18: 767–778. 19010793 10.1093/hmg/ddn388 PMC4334814

[62] Nichols WC, Pankratz N, Hernandez D, Paisan-Ruiz C, Jain S, et al (2005) Genetic screening for a single common LRRK2 mutation in familial Parkinson's disease. Lancet 365: 410–412. 15680455 10.1016/S0140-6736(05)17828-3

[63] Karamohamed S, Golbe LI, Mark MH, Lazzarini AM, Suchowersky O, et al (2005) Absence of previously reported variants in the SCNA (G88C and G209A), NR4A2 (T291D and T245G) and the DJ-1 (T497C) genes in familial Parkinson's disease from the GenePD study. Mov Disord 20: 1188–1191. 15966003 10.1002/mds.20515

[64] Helms C, Cao L, Krueger JG, Wijsman EM, Chamian F, et al (2003) A putative RUNX1 binding site variant between SLC9A3R1 and NAT9 is associated with susceptibility to psoriasis. Nat Genet 35: 349–356. 14608357 10.1038/ng1268

[65] Nair RP, Stuart PE, Nistor I, Hiremagalore R, Chia NV, et al (2006) Sequence and haplotype analysis supports HLA-C as the psoriasis susceptibility 1 gene. Am J Hum Genet 78: 827–851. 16642438 10.1086/503821 PMC1474031

[66] Nair RP, Duffin KC, Helms C, Ding J, Stuart PE, et al (2009) Genome-wide scan reveals association of psoriasis with IL-23 and NF-kappaB pathways. Nat Genet 41: 199–204. 19169254 10.1038/ng.311 PMC2745122

[67] Suarez BK, Duan J, Sanders AR, Hinrichs AL, Jin CH, et al (2006) Genomewide linkage scan of 409 European-ancestry and African American families with schizophrenia: suggestive evidence of linkage at 8p23.3-p21.2 and 11p13.1-q14.1 in the combined sample. Am J Hum Genet 78: 315–333. 16400611 10.1086/500272 PMC1380238

[68] Schizophrenia Psychiatric Genome-Wide Association Study (GWAS) Consortium (2011) Genome-wide association study identifies five new schizophrenia loci. Nat Genet 43: 969–976. 21926974 10.1038/ng.940 PMC3303194

[69] Harley JB, Alarcon-Riquelme ME, Criswell LA, Jacob CO, Kimberly RP, et al (2008) Genome-wide association scan in women with systemic lupus erythematosus identifies susceptibility variants in ITGAM, PXK, KIAA1542 and other loci. Nat Genet 40: 204–210. 18204446 10.1038/ng.81 PMC3712260

[70] Hom G, Graham RR, Modrek B, Taylor KE, Ortmann W, et al (2008) Association of systemic lupus erythematosus with C8orf13-BLK and ITGAM-ITGAX. New Engl J Med 358: 900–909. 18204098 10.1056/NEJMoa0707865

[71] Scott LJ, Mohlke KL, Bonnycastle LL, Willer CJ, Li Y, et al (2007) A genome-wide association study of type 2 diabetes in Finns detects multiple susceptibility variants. Science 316: 1341–1345. 17463248 10.1126/science.1142382 PMC3214617

[72] Saxena R, Voight BF, Lyssenko V, Burtt NP, de Bakker PI, et al (2007) Genome-wide association analysis identifies loci for type 2 diabetes and triglyceride levels. Science 316: 1331–1336. 17463246 10.1126/science.1142358

[73] Barrett JC, Lee JC, Lees CW, Prescott NJ, Anderson CA, et al (2009) Genome-wide association study of ulcerative colitis identifies three new susceptibility loci, including the HNF4A region. Nat Genet 41: 1330–1334. 19915572 10.1038/ng.483 PMC2812019

[74] Jin Y, Birlea SA, Fain PR, Gowan K, Riccardi SL, et al (2010) Variant of TYR and autoimmunity susceptibility loci in generalized vitiligo. New Engl J Med 362: 1686–1697. 20410501 10.1056/NEJMoa0908547 PMC2891985

[75] Jin Y, Birlea SA, Fain PR, Ferrara TM, Ben S, et al (2012) Genome-wide association analyses identify 13 new susceptibility loci for generalized vitiligo. Nat Genet 44: 676–680. 22561518 10.1038/ng.2272 PMC3366044

[76] Ahn R, Ding YC, Murray J, Fasano A, Green PH, et al (2012) Association analysis of the extended MHC region in celiac disease implicates multiple independent susceptibility loci. PLoS One 7: e36926. 22615847 10.1371/journal.pone.0036926 PMC3355177

[77] Purcell S, Neale B, Toddbrown K, Thomas L, Ferreira M, et al (2007) PLINK: A Tool Set for Whole-Genome Association and Population-Based Linkage Analyses. Am J Hum Genet 81: 559–575. 17701901 10.1086/519795 PMC1950838

[78] Patterson N, Price AL, Reich D (2006) Population structure and eigenanalysis. PLoS Genet 2: e190. 17194218 10.1371/journal.pgen.0020190 PMC1713260

[79] Kendler KS, Diehl SR (1993) The genetics of schizophrenia: a current, genetic-epidemiologic perspective. Schizophrenia Bull 19: 261–285. 10.1093/schbul/19.2.261 8322035

[80] Cunningham-Rundles C (2001) Physiology of IgA and IgA deficiency. J Clin Immunol 21: 303–309. 11720003 10.1023/a:1012241117984

[81] Macpherson A, Khoo UY, Forgacs I, Philpott-Howard J, Bjarnason I (1996) Mucosal antibodies in inflammatory bowel disease are directed against intestinal bacteria. Gut 38: 365–375. 8675088 10.1136/gut.38.3.365 PMC1383064

[82] Bouvet JP, Fischetti VA (1999) Diversity of antibody-mediated immunity at the mucosal barrier. Infect Immun 67: 2687–2691. 10338470 10.1128/iai.67.6.2687-2691.1999 PMC96571

[83] Rubino SJ, Selvanantham T, Girardin SE, Philpott DJ (2012) Nod-like receptors in the control of intestinal inflammation. Curr Opin Immunol 24: 398–404. 22677577 10.1016/j.coi.2012.04.010

[84] Pearlman DS (1999) Pathophysiology of the inflammatory response. J Allergy Clin Immunol 104: S132–137. 10518809 10.1016/s0091-6749(99)70308-8

[85] Ventriglia M, Bocchio Chiavetto L, Benussi L, Binetti G, Zanetti O, et al (2002) Association between the BDNF 196 A/G polymorphism and sporadic Alzheimer's disease. Mol Psychiatry 7: 136–137. 11840305 10.1038/sj.mp.4000952

[86] Momose Y, Murata M, Kobayashi K, Tachikawa M, Nakabayashi Y, et al (2002) Association studies of multiple candidate genes for Parkinson's disease using single nucleotide polymorphisms. Ann Neurol 51: 133–136. 11782995 10.1002/ana.10079

[87] Sen S, Nesse RM, Stoltenberg SF, Li S, Gleiberman L, et al (2003) A BDNF coding variant is associated with the NEO personality inventory domain neuroticism, a risk factor for depression. Neuropsychopharmacology 28: 397–401. 12589394 10.1038/sj.npp.1300053

[88] Berndt SI, Gustafsson S, Magi R, Ganna A, Wheeler E, et al (2013) Genome-wide meta-analysis identifies 11 new loci for anthropometric traits and provides insights into genetic architecture. Nat Genet 45: 501–512. 23563607 10.1038/ng.2606 PMC3973018

[89] Novembre J, Johnson T, Bryc K, Kutalik Z, Boyko AR, et al (2008) Genes mirror geography within Europe. Nature 456: 98–101. 18758442 10.1038/nature07331 PMC2735096

[90] Price AL, Patterson NJ, Plenge RM, Weinblatt ME, Shadick NA, et al (2006) Principal components analysis corrects for stratification in genome-wide association studies. Nat Genet 38: 904–909. 16862161 10.1038/ng1847

[91] Klei L, Luca D, Devlin B, Roeder K (2008) Pleiotropy and principal components of heritability combine to increase power for association analysis. Genet Epidemiol 32: 9–19. 17922480 10.1002/gepi.20257

[92] Glocker E, Grimbacher B (2012) Inflammatory bowel disease: is it a primary immunodeficiency? Cell Mol Life Sci 69: 41–48. 21997382 10.1007/s00018-011-0837-9 PMC11114923

[93] Hayee B, Rahman FZ, Sewell G, Smith AM, Segal AW (2010) Crohn's disease as an immunodeficiency. Expert Rev Clin Immunol 6: 585–596. 20594132 10.1586/eci.10.32 PMC4618314

[94] Durany N, Michel T, Zochling R, Boissl KW, Cruz-Sanchez FF, et al (2001) Brain-derived neurotrophic factor and neurotrophin 3 in schizophrenic psychoses. Schizophr Res 52: 79–86. 11595394 10.1016/s0920-9964(00)00084-0

[95] Buckley PF, Mahadik S, Pillai A, Terry A (2007) Neurotrophins and schizophrenia. Schizophr Res 94: 1–11. 17524622 10.1016/j.schres.2007.01.025

[96] Gusev A, Bhatia G, Zaitlen N, Vilhjalmsson BJ, Diogo D, et al (2013) Quantifying missing heritability at known GWAS loci. PLoS Genet 9: e1003993. 24385918 10.1371/journal.pgen.1003993 PMC3873246

