# Supplementary figures and images for: Principal Component Analysis Characterizes Shared Pathogenetics from Genome-Wide Association Studies
Source: PLoS Comput Biol. 2014 Sep 11;10(9):e1003820. doi: 10.1371/journal.pcbi.1003820 (PMC4161298; doi:10.1371/journal.pcbi.1003820)

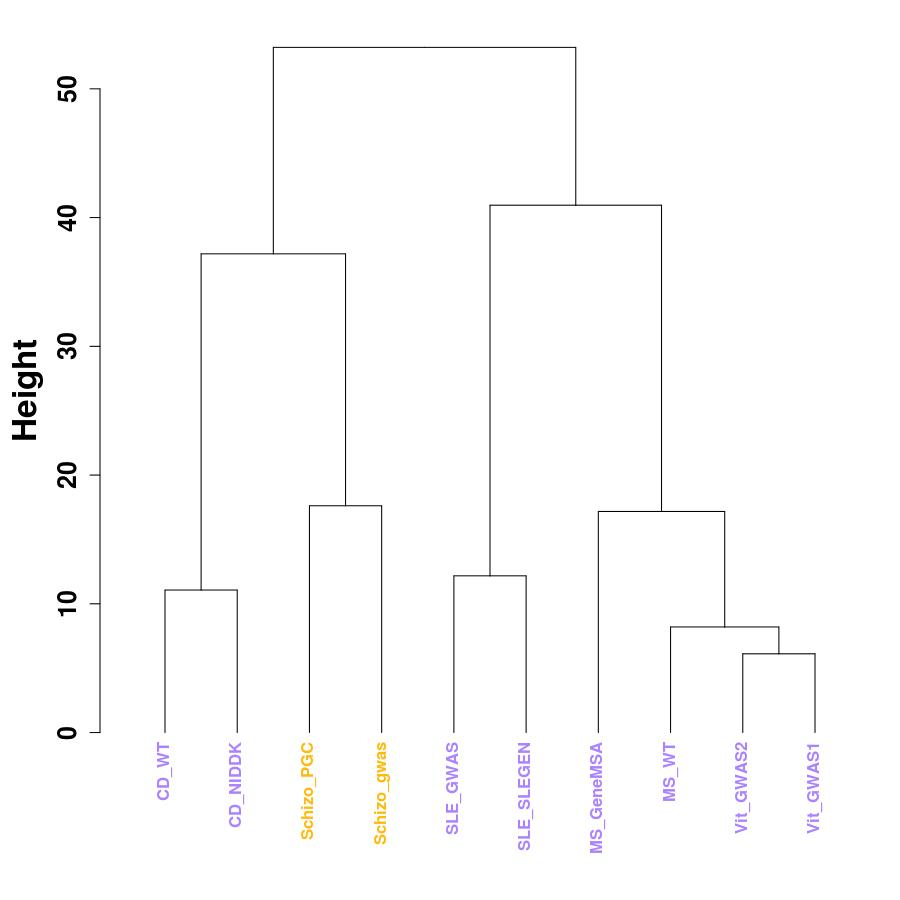

Supplement: Figure S1 — Clustering dendrogram of datasets of the same diseases using physical distance mapping. SNPs were mapped to genes if they were within 10 kb of the gene. Clustering analysis of resulting disPCA revealed the same clusters as disPCA with genetic coordinates (Figure 3). (TIFF) [file pcbi.1003820.s001.tiff]

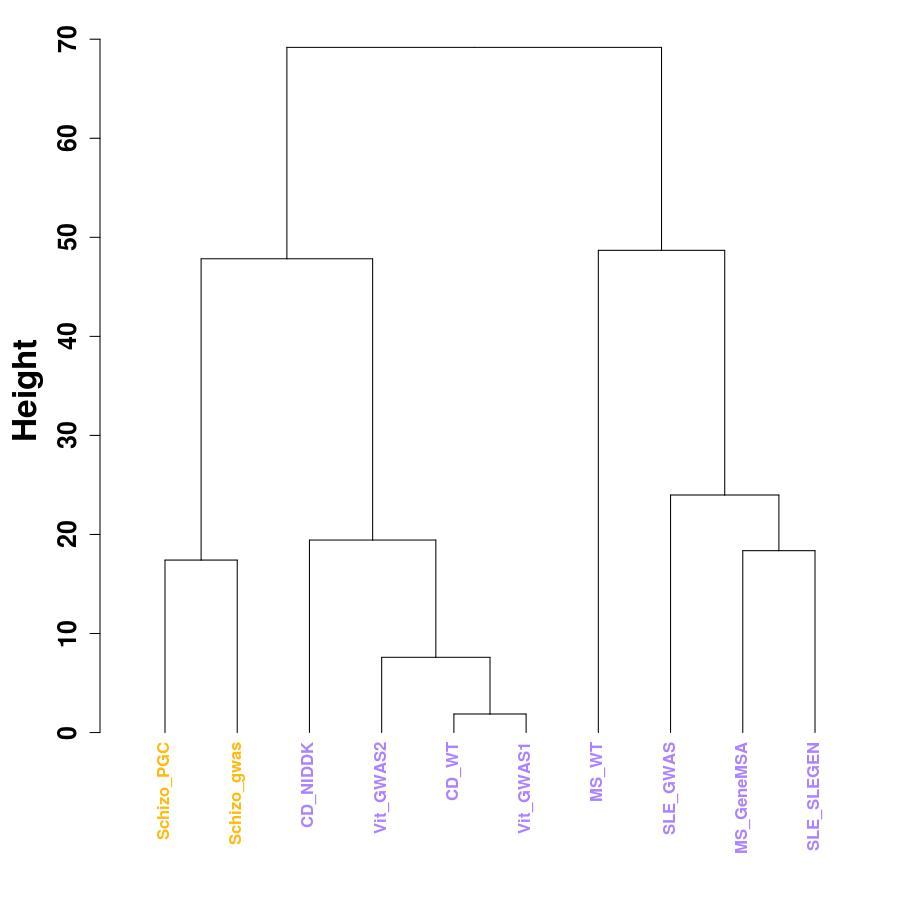

Supplement: Figure S2 — Clustering dendrogram of datasets of the same diseases with the truncated product method. Similar to Figure 3, with the truncated product method used to combine SNP p-values per gene. (TIFF) [file pcbi.1003820.s002.tiff]

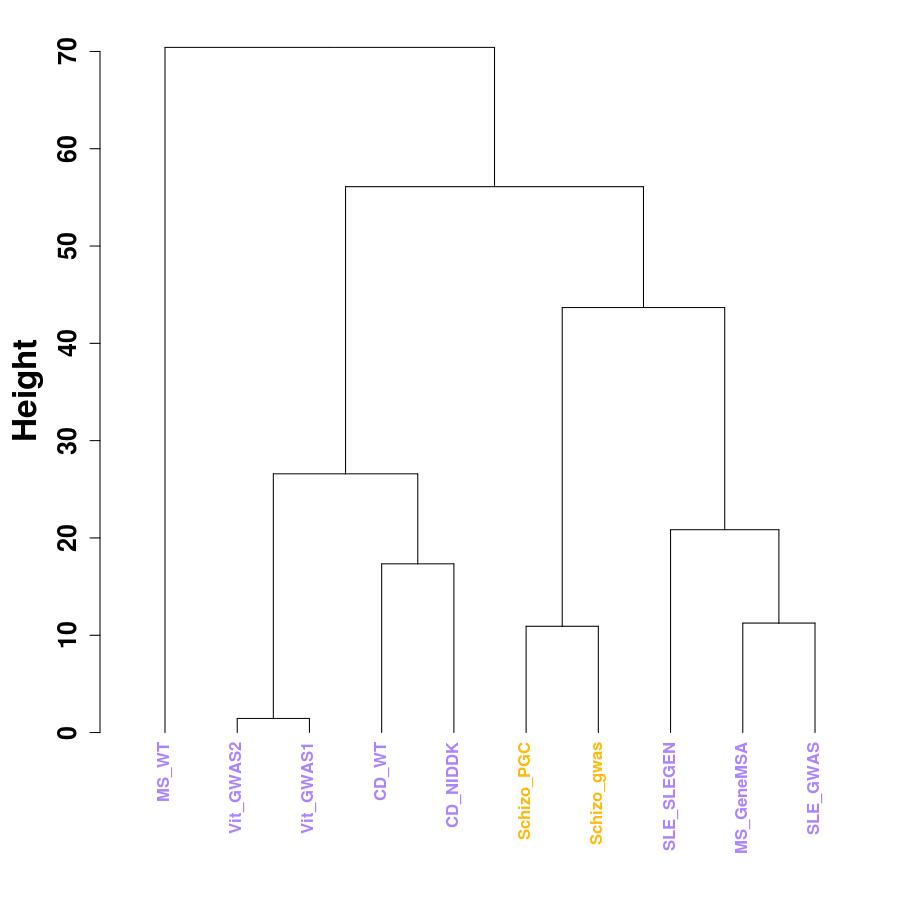

Supplement: Figure S3 — Clustering dendrogram of datasets of the same diseases with truncated tail strength method. Similar to Figure 3, with the truncated tail strength method used to combine SNP p-values per gene. (TIFF) [file pcbi.1003820.s003.tiff]

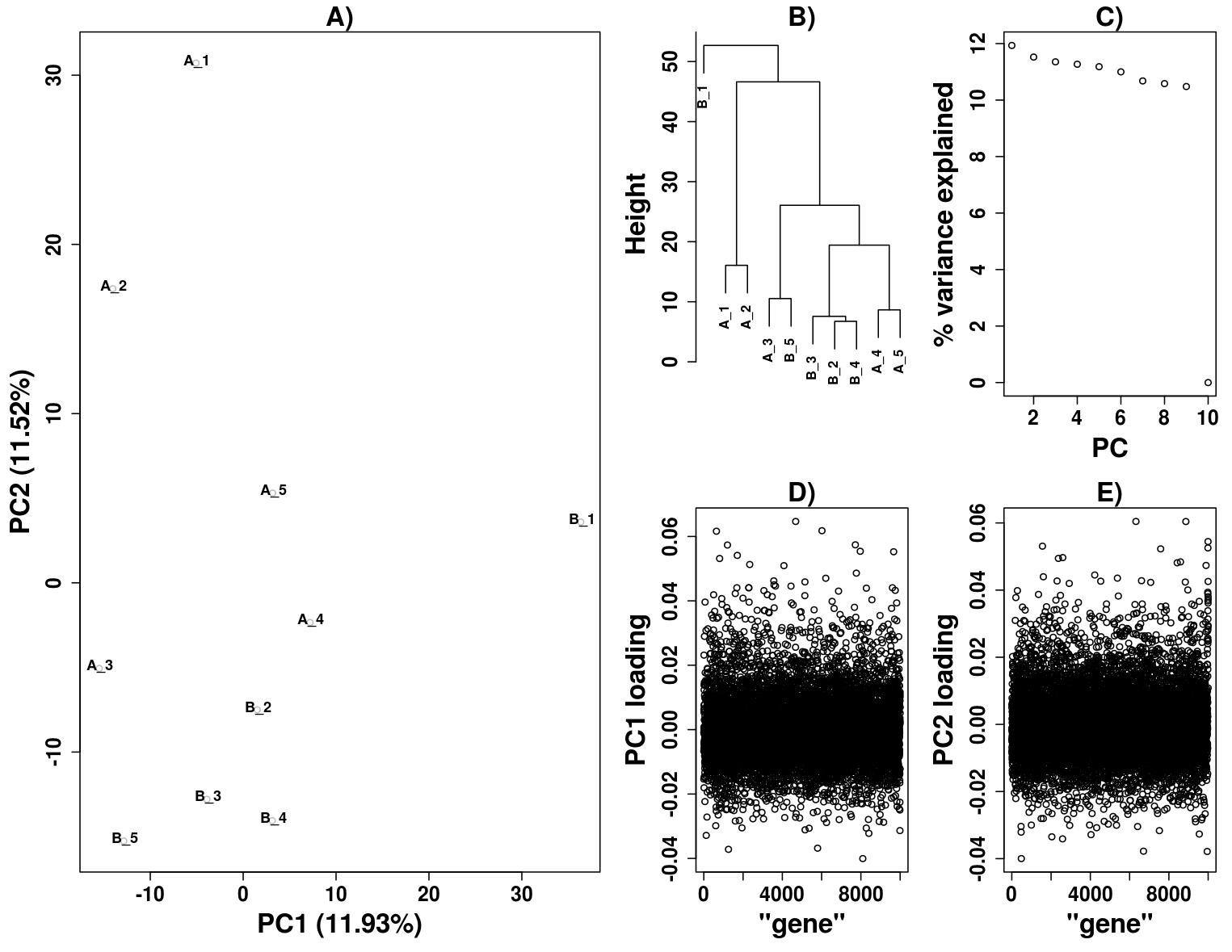

Supplement: Figure S4 — Simulated diseases with ten nominally significant genes. A) Similar to Figure 1 in main text with only ten nominally significant genes for each set of pleiotropic diseases (Materials and Methods). Clustering of the diseases sets is not observed. B) Clustering dendrogram as similarly presented in Figure 1b. C) The portion of variance explained by each PC is displayed. D–E) The loadings for PC1 and PC2 are displayed. (TIFF) [file pcbi.1003820.s004.tiff]

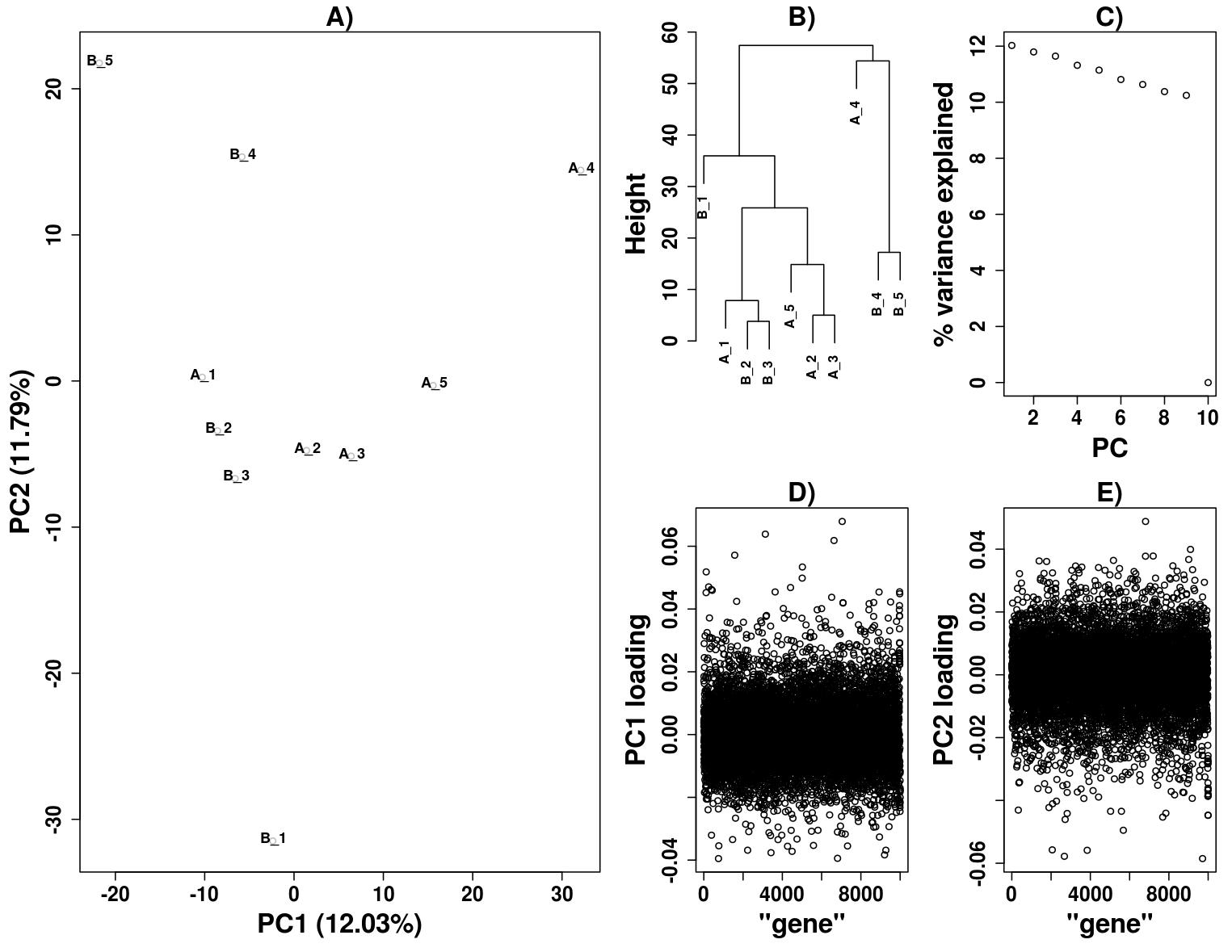

Supplement: Figure S5 — Simulated diseases with twenty nominally significant genes. A) Similar to Figure 1 with twenty nominally significant genes for each set of pleiotropic diseases. As in Figure S2, diseases are not clearly clustering according to the sets though nominally significant genes are enriched for larger absolute loadings (Materials and Methods). B) Clustering dendrogram as similarly presented in Figure 1b. C) The portion of variance explained by each PC is displayed. D–E) The loadings for PC1 and PC2 are displayed. (TIFF) [file pcbi.1003820.s005.tiff]

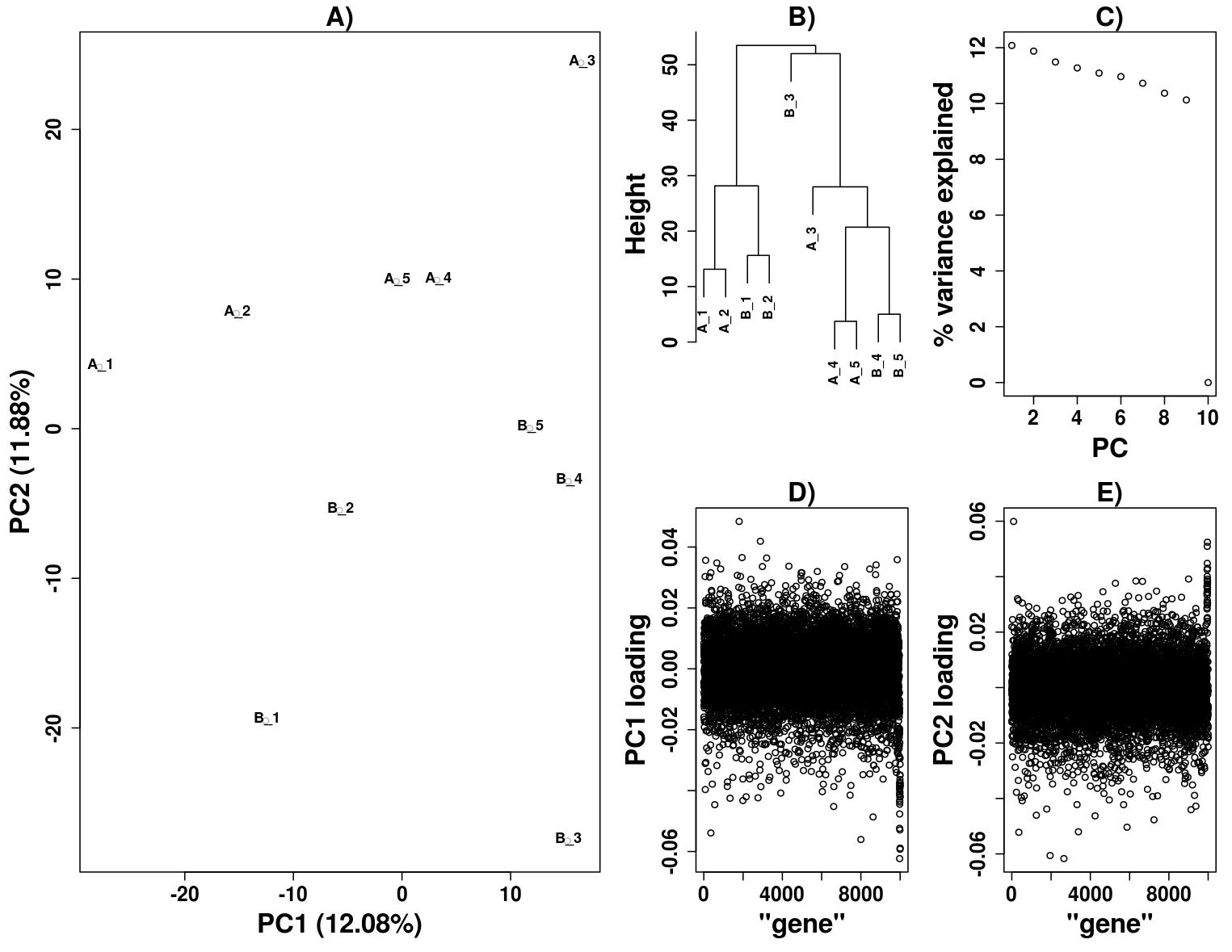

Supplement: Figure S6 — Simulated diseases with thirty nominally significant genes. A) Similar to Figure 1 with thirty nominally significant genes for each set of pleiotropic diseases. The proper clustering of diseases is beginning to emerge. B) Clustering dendrogram as similarly presented in Figure 1b. C) The portion of variance explained by each PC is displayed. D–E) The loadings for PC1 and PC2 are displayed. Genes with nominally significant p-values are enriched for larger absolute loadings. (TIFF) [file pcbi.1003820.s006.tiff]

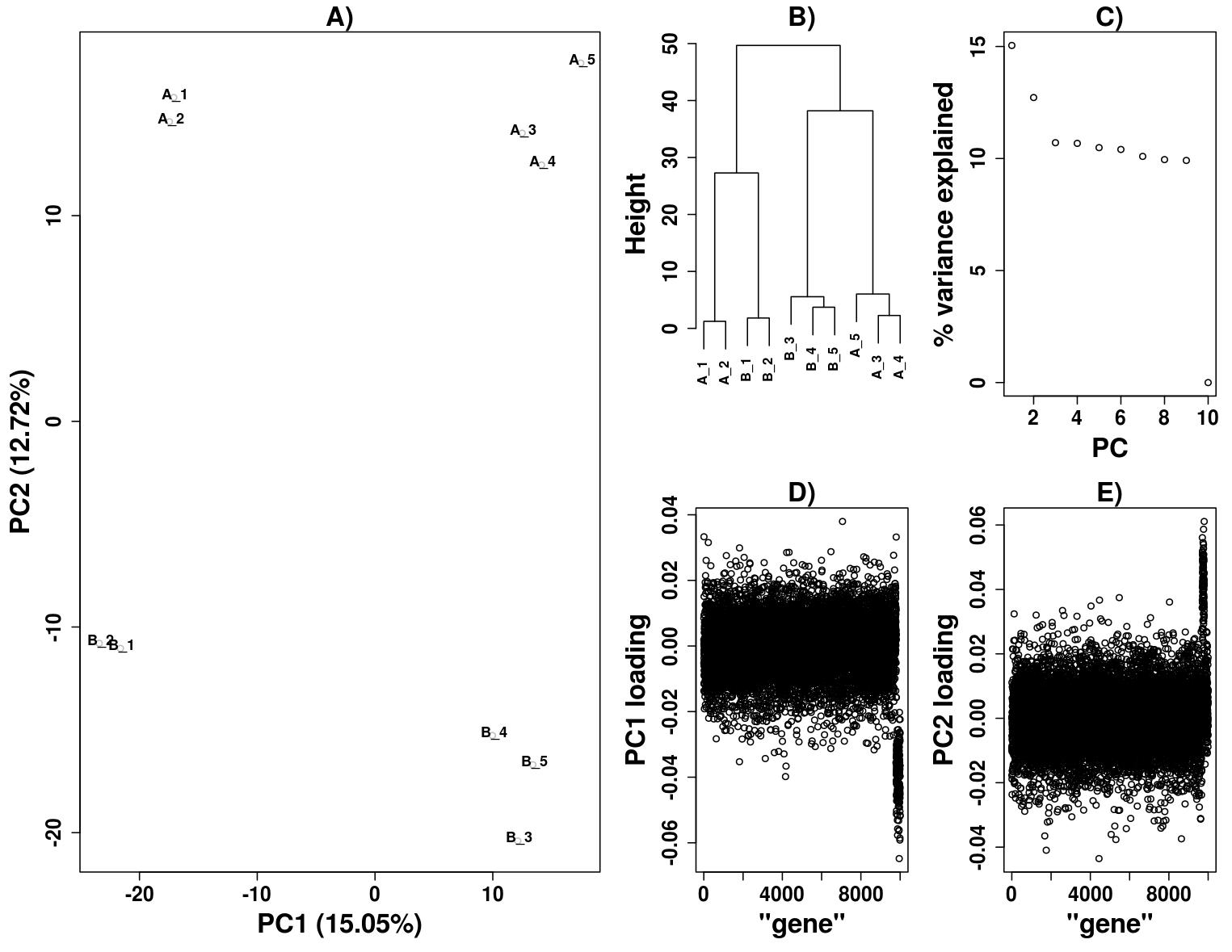

Supplement: Figure S7 — Simulated diseases with 100 and 200 nominally significant genes. A) Similar to Figure 1 with 100 and 200 nominally significant genes for the two sets of pleiotropic diseases. Disease sets are tightly clustered and the first two PCs explain a larger portion of the variance compared to other PCs. B) Clustering dendrogram as similarly presented in Figure 1b. C) The portion of variance explained by each PC is displayed. D–E) The loadings for PC1 and PC2 are displayed. (TIFF) [file pcbi.1003820.s007.tiff]

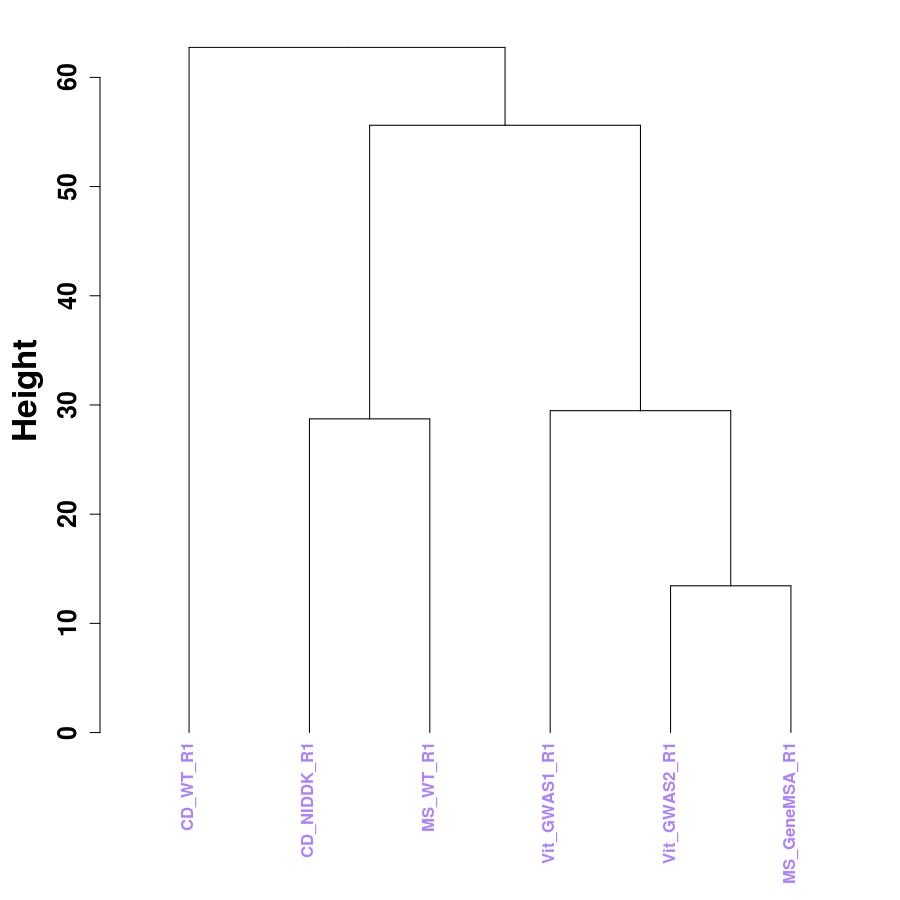

Supplement: Figure S8 — Clustering dendrogram of Replication Set 1 datasets. Clustering of the distance in PC space between datasets in Replication Set 1. Diseases include vitiligo (Vit), multiple sclerosis (MS), schizophrenia (Schizo) and Crohn's disease (CD). (TIFF) [file pcbi.1003820.s008.tiff]

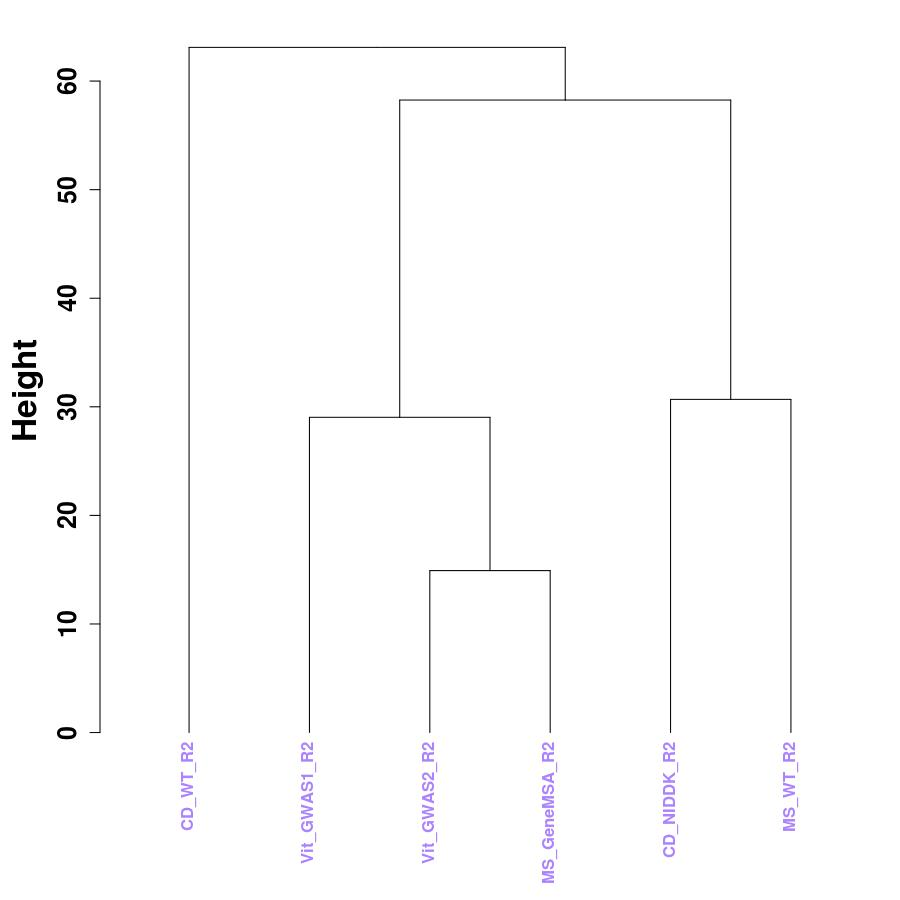

Supplement: Figure S9 — Clustering dendrogram of Replication Set 2 datasets. Similar to Figure S8 with datasets from Replication Set 2. (TIFF) [file pcbi.1003820.s009.tiff]

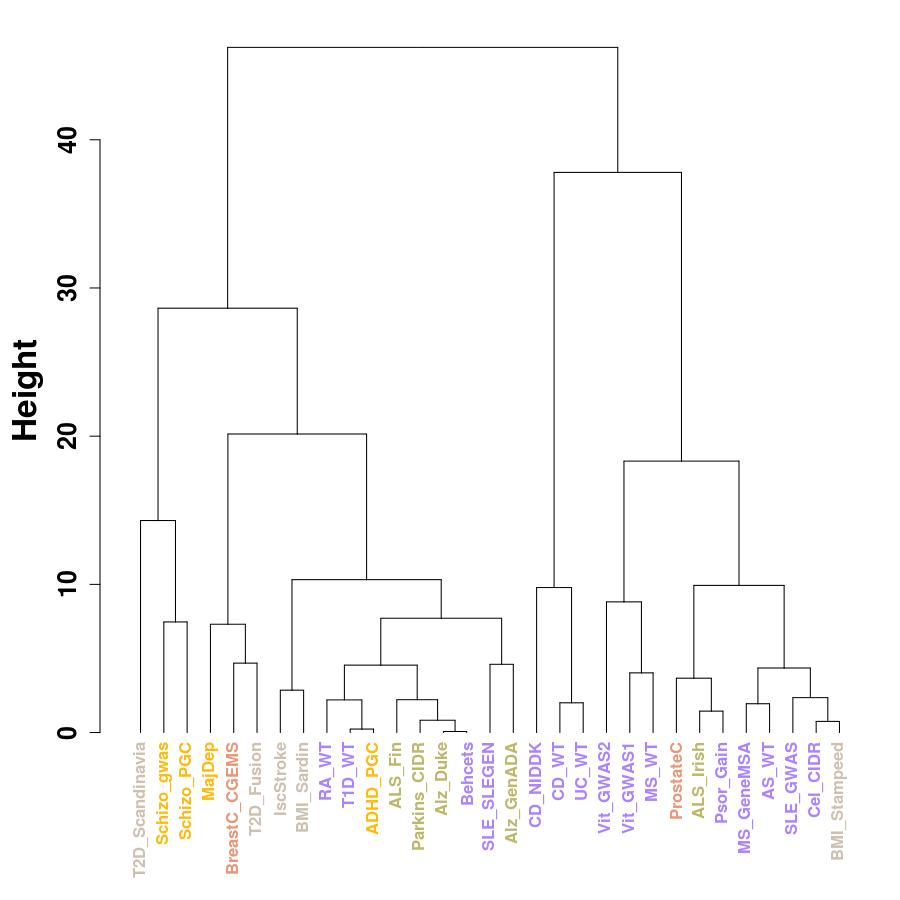

Supplement: Figure S10 — Clustering dendrogram of all diseases and traits excluding the HLA and surrounding regions. Figure is similar to Figure 5, with clustering analysis of distance between datasets based on the disPCA between all diseases and traits presented in Table S2 after removing the HLA and surrounding regions. (TIFF) [file pcbi.1003820.s010.tiff]

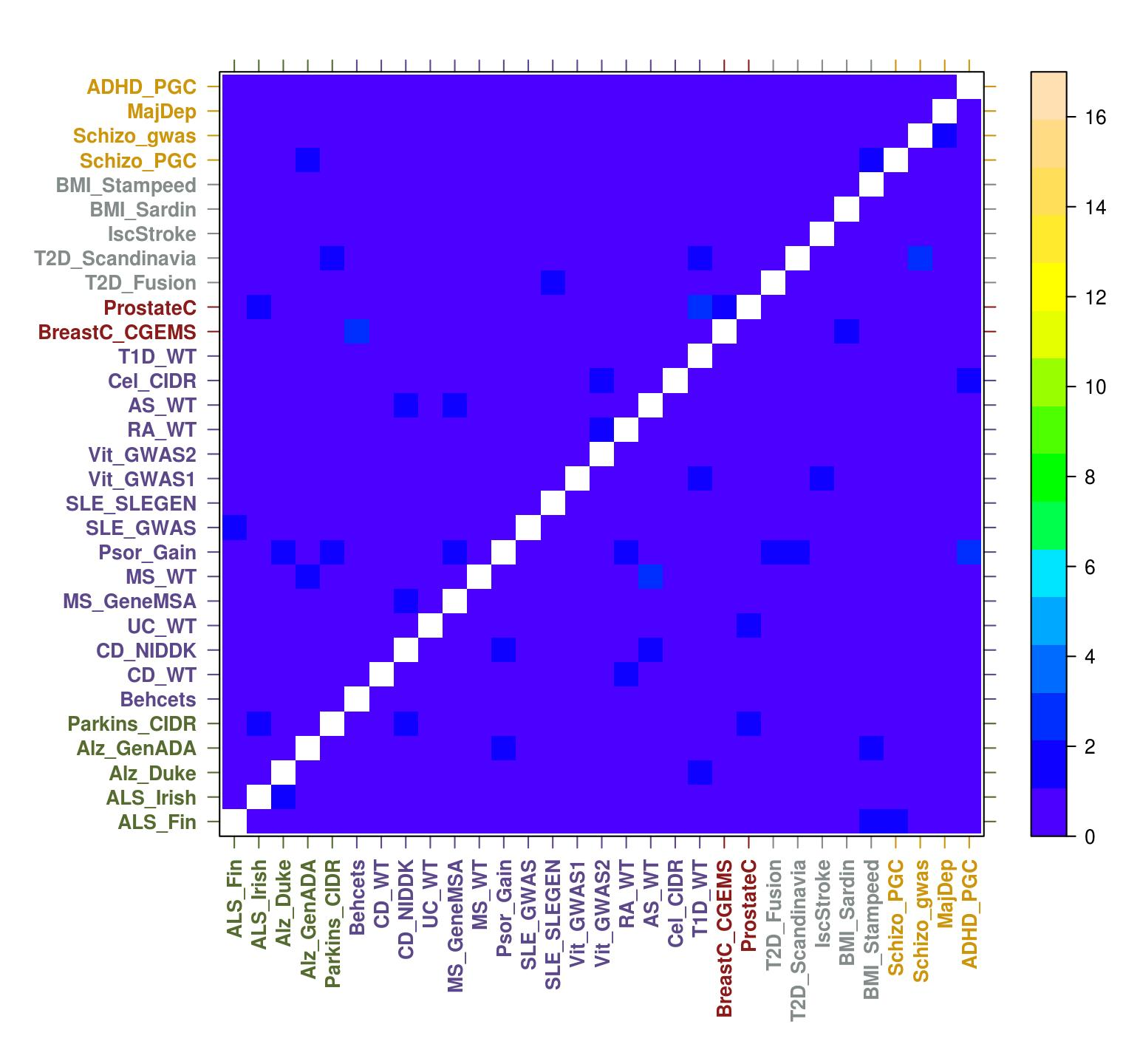

Supplement: Figure S11 — Non-random distribution of randomly chosen genes. A random subset of genes were chosen to be tested for non-random distribution in diseases on the x-axis, with −log10 presented on the color scale on the right. White entries denote p-values<1×10^−17^. (TIFF) [file pcbi.1003820.s011.tiff]

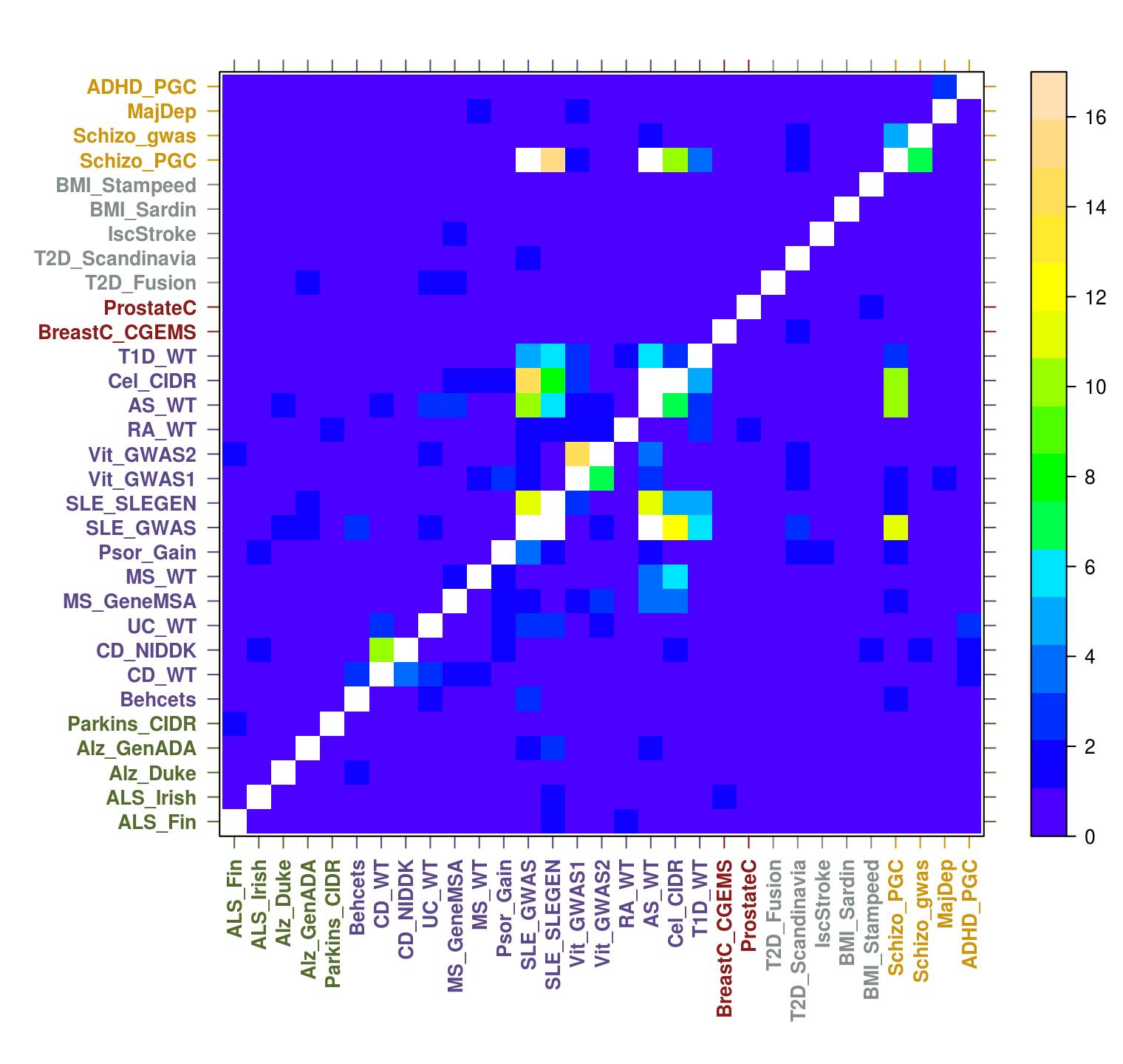

Supplement: Figure S12 — Non-random distribution for distance pruned set of genes. Genes were filtered such that no two genes were within 0.1 cM of another. The remaining subset of genes was then tested for non-random distribution in diseases on the x-axis. The −log10 of the p-value is presented on the color scale and white entries denote p-values<1×10^−17^. Results are largely similar to the original without filtering of nearby genes. (TIFF) [file pcbi.1003820.s012.tiff]

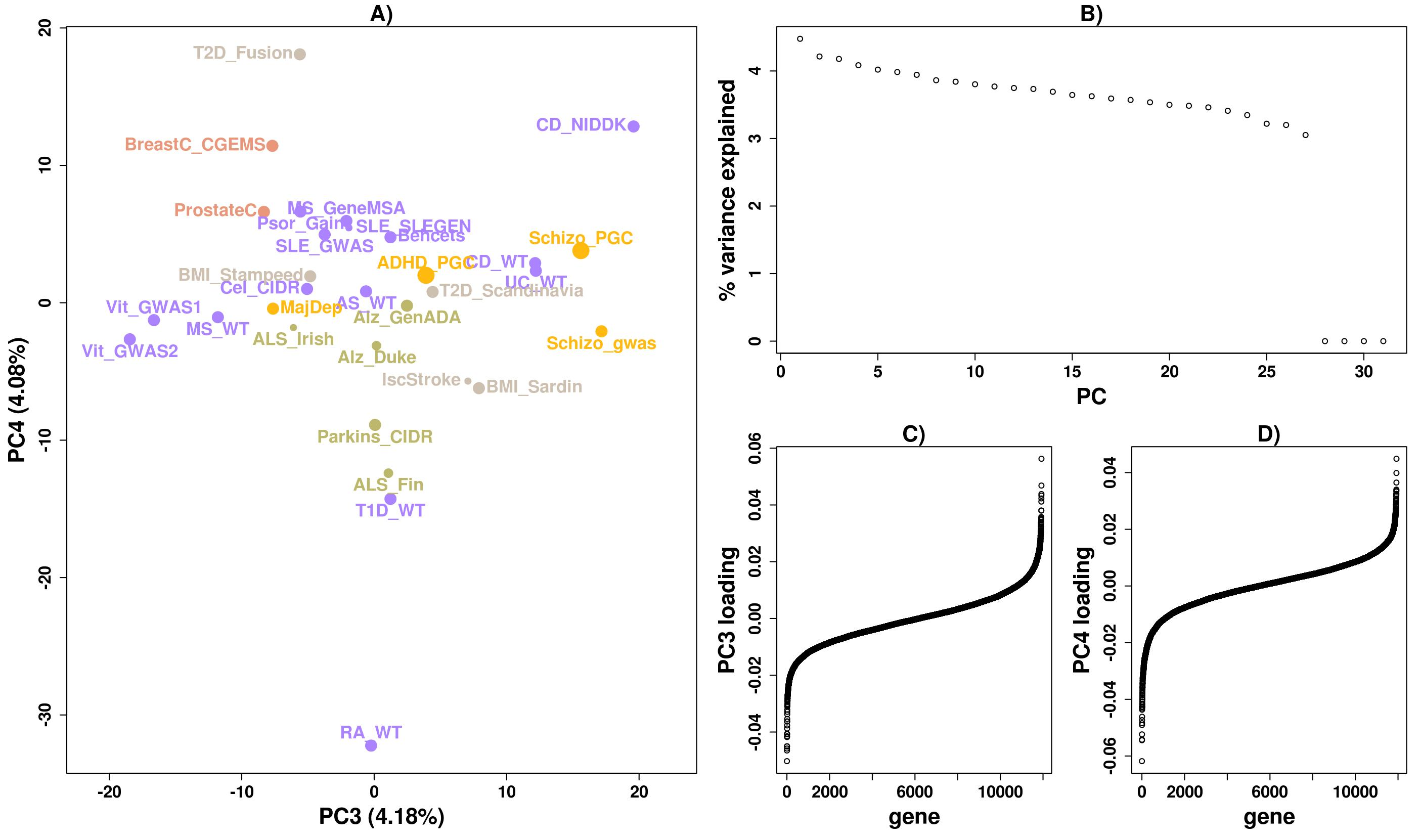

Supplement: Figure S13 — PC3 and PC4 of all diseases disPCA . Similar to Figure 4 with data being presented for PC3 and PC4. A) PC1 accounts for 4.18% of the variance, while PC2 accounts for 4.08%. PC1 clusters schizophrenia and vitiligo datasets together on the two extremes, while PC2 separates rheumatoid arthritis from other diseases and traits. B) The portion of variance explained by each PC is displayed. C) The weightings for genes on PC1 are displayed and ordered according to their weights. D) Similar to (C) where loadings are for PC2. (TIFF) [file pcbi.1003820.s013.tiff]
