# Supplementary material for: Principal Component Analysis Characterizes Shared Pathogenetics from Genome-Wide Association Studies
Source: PLoS Comput Biol. 2014 Sep 11;10(9):e1003820. doi: 10.1371/journal.pcbi.1003820 (PMC4161298; doi:10.1371/journal.pcbi.1003820)
Supplement: Table S1 — Comparison of loadings between disPCA with mapping based on physical or genetic coordinates. Loadings for the top 50 genes ranked by either a physical or genetic coordinates based disPCA were compared. ‘Correlation’ denotes the Pearson's correlation coefficient with its significance denoted in the ‘p-value’ column. Rows denoted by ‘mean(PC1,PC2)’ indicate the correlation between the 50 genes with the largest average loading of PC1 and PC2. (DOC) [file pcbi.1003820.s014.doc]

| **Pairs of datasets of the same disease** | | | |
| --- | --- | --- | --- |
| **PC** | **Ranked by** | **Correlation** | **p-value** |
| **1** | Physical | 0.62 | 1.7x10^-6^ |
|  | Genetic | 0.69 | 3.6x10^-8^ |
| **2** | Physical | 0.51 | 1.0x10^-4^ |
|  | Genetic | 0.31 | 0.0287 |
| **mean(PC1,PC2)** | Physical | 0.74 | 1.1x10^-9^ |
|  | Genetic | 0.67 | 8.4x10^-8^ |

**Table S1. Comparison of loadings between *disPCA* with mapping based on physical or genetic coordinates.** Loadings for the top 50 genes ranked by either a physical or genetic coordinates based *disPCA* were compared. ‘Correlation’ denotes the Pearson’s correlation coefficient with its significance denoted in the ‘p-value’ column. Rows denoted by ‘mean(PC1,PC2)’ indicate the correlation between the 50 genes with the largest average loading of PC1 and PC2.
