# Supplementary material for: Principal Component Analysis Characterizes Shared Pathogenetics from Genome-Wide Association Studies
Source: PLoS Comput Biol. 2014 Sep 11;10(9):e1003820. doi: 10.1371/journal.pcbi.1003820 (PMC4161298; doi:10.1371/journal.pcbi.1003820)
Supplement: Table S2 — Dataset attributes. Various attributes of datasets utilized in this study. (DOC) [file pcbi.1003820.s015.doc]

| **Study Name** | **Disease** | **Obtained via** | **Association Method** | **Array** | **Sample Size** |
| --- | --- | --- | --- | --- | --- |
| ALS Finland *(ALS_Fin)* | ALS | dbGaP | Logistic regression | Overlap between Illumina 1M and CNV 370 | 973 |
| ALS Irish*(ALS_Irish)* | ALS | dbGaP | Logistic regression | Illumina 550k | 432 |
| Duke Alzheimer's *(Alz_Duke)* | Alzheimer's Disease | http://humangenome.duke.edu/available-datasets | Logistic regression | Illumina 550 | 699 |
| GenADA *(Alz_GenADA)* | Alzheimer's Disease | dbGaP | Logistic regression | Affymetrix 400 | 1588 |
| WTCCC2 AS (*AS_WT)* | Ankyolosing Spondylitis | WTCCC | Logistic regression | Illumina 1M | 2732 |
| ADHD PGC (*ADHD_PGC)* | ADHD | PGC | Meta-analysis | Imputation | 5415 |
| Behcet's GWAS *(Behcets_GWAS)* | Bechet's | dbGaP | Chis-sq | Illumina CNV 370 | 2493 |
| BMI Stampeed (*BMI_Stampeed)* | BMI | dbGaP | Linear regression | Illumina CNV 370 | 5415 |
| BMI Sardinia (*BMI_Sardin)* | BMI | dbGaP | Merlin | Affymetrix 500 | 1412 |
| CGEMS Breast Cancer *(BreastC_CGEMS)* | Breast Cancer | dbGaP | Logistic regression | Illumina 550 | 2287 |
| CIDR Celiac *(CeliacD_CIDR)* | Celiac disease | dbGaP | Logistic regression | Illumina 660 | 2246 |
| NIDDK IBD *(CD_NIDDK)* | Crohn's disease | dbGaP | Chis-sq | Illumina 300 | 1028 |
| WTCCC CD *(CD_WTCCC)* | Crohn's disease | WTCCC | Logistic regression | Affymetrix 500 | 3293 |
| Ischemic Stroke (*IscStroke)* | Ischemic Stroke | dbGaP | Logistic regression | Illumina 300 | 485 |
| Major Depression GWAS (*MajDep)* | Major depression | dbGaP | Logistic regression | Perlgen 600k | 3741 |
| WTCCC2 MS (*MS_WT)* | Multiple Sclerosis | WTCCC | Logistic regression | Illumina 1M | 4055 |
| GeneMSA *(MS_GeneMSA)* | Multiple Sclerosis | dbGaP | Logistic Regression | Illumina 550 | 2000 |
| CIDR Parkinson's *(Parkin_CIDR)* | Parkinson's | dbGaP | Logistic regression | Illumina CNV 370 | 1991 |
| CASP *(Psor_CASP)* | Psoriasis | dbGaP | Chi-sq | Perlgen 600k | 2825 |
| WTCCC RA *(RA_WTCCC)* | Rheumatoid arthritis | WTCCC | Logistic regression | Affymetrix 500 | 3481 |
| Schizophrenia GWAS *(Schizo_GWAS)* | Schizophrenia | dbGaP | Chi-sq | Affymetrix 6.0 | 2659 |
| PGC Schizophrenia *(Schizo_PGC)* | Schizophrenia | PGC | Meta-analysis | Imputation | 21,856 |
| SLEGEN *(SLE_SLEGEN)* | SLE | dbGaP | additive model | Illumina 300 | 297 |
| SLE GWAS *(SLE_GWAS)* | SLE | dbGaP | Chi-sq | Illumina 550 | 4651 |
| T2D Fusion *(T2D_Fusion)* | T2D | dbGaP | Logistic regression | Illumina 300 | 1706 |
| T2D Scandinavia *(T2D_Scandinavia)* | T2D | http://www.broadinstitute.org/diabetes/scandinavs/type2.html | Cochran-Mantel-Haenszel | Affymetrix 500 | 3000 |
| WTCCC2 UC (*UC_WT)* | Ulcerative colitis | WTCCC | Logistic regression | Affymetrix 6.0 | 404 |
| VitGene *(Vit_GWAS1)* | Vitiligo | dbGaP | Logistic regression | Illumina 610 | 4327 |
| Vitiligo GWAS2 *(Vit_GWAS2)* | Vitiligo | Collaboration | Logistic regression | Illumina 660 | 3632 |

**Table S2. Dataset attributes.** Various attributes of datasets utilized in this study.

**REFERENCES**

1. Laaksovirta H, Peuralinna T, Schymick JC, Scholz SW, Lai SL, et al. (2010) Chromosome 9p21 in amyotrophic lateral sclerosis in Finland: a genome-wide association study. Lancet Neurol 9: 978-985.

2. Cronin S, Berger S, Ding J, Schymick JC, Washecka N, et al. (2008) A genome-wide association study of sporadic ALS in a homogenous Irish population. Hum Mol Genet 17: 768-774.

3. Heinzen EL, Need AC, Hayden KM, Chiba-Falek O, Roses AD, et al. (2010) Genome-wide scan of copy number variation in late-onset Alzheimer's disease. J Alzheimers Dis 19: 69-77.

4. Li H, Wetten S, Li L, St Jean PL, Upmanyu R, et al. (2008) Candidate single-nucleotide polymorphisms from a genomewide association study of Alzheimer disease. Arch Neurol 65: 45-53.

5. Evans DM, Spencer CC, Pointon JJ, Su Z, Harvey D, et al. (2011) Interaction between ERAP1 and HLA-B27 in ankylosing spondylitis implicates peptide handling in the mechanism for HLA-B27 in disease susceptibility. Nat Genet 43: 761-767.

6. Neale BM, Medland SE, Ripke S, Asherson P, Franke B, et al. (2010) Meta-analysis of genome-wide association studies of attention-deficit/hyperactivity disorder. J Am Acad Child Adolesc Psychiatry 49: 884-897.

7. Remmers EF, Cosan F, Kirino Y, Ombrello MJ, Abaci N, et al. (2010) Genome-wide association study identifies variants in the MHC class I, IL10, and IL23R-IL12RB2 regions associated with Behcet's disease. Nat Genet 42: 698-702.

8. Sabatti C, Service SK, Hartikainen AL, Pouta A, Ripatti S, et al. (2009) Genome-wide association analysis of metabolic traits in a birth cohort from a founder population. Nat Genet 41: 35-46.

9. Scuteri A, Sanna S, Chen WM, Uda M, Albai G, et al. (2007) Genome-wide association scan shows genetic variants in the FTO gene are associated with obesity-related traits. PLoS Genet 3: e115.

10. Hunter DJ, Kraft P, Jacobs KB, Cox DG, Yeager M, et al. (2007) A genome-wide association study identifies alleles in FGFR2 associated with risk of sporadic postmenopausal breast cancer. Nat Genet 39: 870-874.

11. Ahn R, Ding YC, Murray J, Fasano A, Green PH, et al. (2012) Association analysis of the extended MHC region in celiac disease implicates multiple independent susceptibility loci. PLoS One 7: e36926.

12. Duerr RH, Taylor KD, Brant SR, Rioux JD, Silverberg MS, et al. (2006) A genome-wide association study identifies IL23R as an inflammatory bowel disease gene. Science 314: 1461-1463.

13. ‎The Wellcome Trust Case Control Consortium (2007) Genome-wide association study of 14,000 cases of seven common diseases and 3,000 shared controls. Nature 447: 661-678.

14. Matarin M, Brown WM, Scholz S, Simon-Sanchez J, Fung HC, et al. (2007) A genome-wide genotyping study in patients with ischaemic stroke: initial analysis and data release. Lancet Neurol 6: 414-420.

15. Boomsma DI, Willemsen G, Sullivan PF, Heutink P, Meijer P, et al. (2008) Genome-wide association of major depression: description of samples for the GAIN Major Depressive Disorder Study: NTR and NESDA biobank projects. Eur J Hum Genet 16: 335-342.

16. Sawcer S, Hellenthal G, Pirinen M, Spencer CC, Patsopoulos NA, et al. (2011) Genetic risk and a primary role for cell-mediated immune mechanisms in multiple sclerosis. Nature 476: 214-219.

17. Baranzini SE, Wang J, Gibson RA, Galwey N, Naegelin Y, et al. (2009) Genome-wide association analysis of susceptibility and clinical phenotype in multiple sclerosis. Hum Mol Genet 18: 767-778.

18. Nichols WC, Pankratz N, Hernandez D, Paisan-Ruiz C, Jain S, et al. (2005) Genetic screening for a single common LRRK2 mutation in familial Parkinson's disease. Lancet 365: 410-412.

19. Karamohamed S, Golbe LI, Mark MH, Lazzarini AM, Suchowersky O, et al. (2005) Absence of previously reported variants in the SCNA (G88C and G209A), NR4A2 (T291D and T245G) and the DJ-1 (T497C) genes in familial Parkinson's disease from the GenePD study. Mov Disord 20: 1188-1191.

20. Helms C, Cao L, Krueger JG, Wijsman EM, Chamian F, et al. (2003) A putative RUNX1 binding site variant between SLC9A3R1 and NAT9 is associated with susceptibility to psoriasis. Nat Genet 35: 349-356.

21. Nair RP, Stuart PE, Nistor I, Hiremagalore R, Chia NV, et al. (2006) Sequence and haplotype analysis supports HLA-C as the psoriasis susceptibility 1 gene. Am J Hum Genet 78: 827-851.

22. Nair RP, Duffin KC, Helms C, Ding J, Stuart PE, et al. (2009) Genome-wide scan reveals association of psoriasis with IL-23 and NF-kappaB pathways. Nat Genet 41: 199-204.

23. Suarez BK, Duan J, Sanders AR, Hinrichs AL, Jin CH, et al. (2006) Genomewide linkage scan of 409 European-ancestry and African American families with schizophrenia: suggestive evidence of linkage at 8p23.3-p21.2 and 11p13.1-q14.1 in the combined sample. Am J Hum Genet 78: 315-333.

24. Schizophrenia Psychiatric Genome-Wide Association Study (GWAS) Consortium (2011) Genome-wide association study identifies five new schizophrenia loci. Nat Genet 43: 969-976.

25. Harley JB, Alarcon-Riquelme ME, Criswell LA, Jacob CO, Kimberly RP, et al. (2008) Genome-wide association scan in women with systemic lupus erythematosus identifies susceptibility variants in ITGAM, PXK, KIAA1542 and other loci. Nat Genet 40: 204-210.

26. Hom G, Graham RR, Modrek B, Taylor KE, Ortmann W, et al. (2008) Association of systemic lupus erythematosus with C8orf13-BLK and ITGAM-ITGAX. New Engl J Med 358: 900-909.

27. Scott LJ, Mohlke KL, Bonnycastle LL, Willer CJ, Li Y, et al. (2007) A genome-wide association study of type 2 diabetes in Finns detects multiple susceptibility variants. Science 316: 1341-1345.

28. Saxena R, Voight BF, Lyssenko V, Burtt NP, de Bakker PI, et al. (2007) Genome-wide association analysis identifies loci for type 2 diabetes and triglyceride levels. Science 316: 1331-1336.

29. Barrett JC, Lee JC, Lees CW, Prescott NJ, Anderson CA, et al. (2009) Genome-wide association study of ulcerative colitis identifies three new susceptibility loci, including the HNF4A region. Nat Genet 41: 1330-1334.

30. Jin Y, Birlea SA, Fain PR, Gowan K, Riccardi SL, et al. (2010) Variant of TYR and autoimmunity susceptibility loci in generalized vitiligo. New Engl J Med 362: 1686-1697.

31. Jin Y, Birlea SA, Fain PR, Ferrara TM, Ben S, et al. (2012) Genome-wide association analyses identify 13 new susceptibility loci for generalized vitiligo. Nat Genet 44: 676-680.
