# Supplementary material for: Principal Component Analysis Characterizes Shared Pathogenetics from Genome-Wide Association Studies
Source: PLoS Comput Biol. 2014 Sep 11;10(9):e1003820. doi: 10.1371/journal.pcbi.1003820 (PMC4161298; doi:10.1371/journal.pcbi.1003820)
Supplement: Table S3 — Comparison of loadings between Replication Sets 1 and 2. Loadings for the top 50 genes ranked by either Replication Set 1 or Replication Set 2 were compared. ‘Correlation’ denotes the Pearson's correlation coefficient with its significance denoted in the ‘p-value’ column. Rows denoted by ‘mean(PC1,PC2)’ indicate the correlation between the 50 genes with the largest average loading of PC1 and PC2. (DOC) [file pcbi.1003820.s016.doc]

| **Replication** | | | |
| --- | --- | --- | --- |
| **PC** | **Ranked by** | **Correlation** | **p-value** |
| **1** | Replication 1 | -0.056 | 0.7 |
|  | Replication 2 | 0.28 | 0.049 |
| **2** | Replication 1 | 0.479 | 4.3x10^-4^ |
|  | Replication 2 | 0.634 | 7.7x10^-7^ |
| **mean(PC1,PC2)** | Replication 1 | 0.444 | 1.2x10^-3^ |
|  | Replication 2 | 0.652 | 7.7x10^-7^ |

**Table S3. Comparison of loadings between Replication Sets 1 and 2.** Loadings for the top 50 genes ranked by either Replication Set 1 or Replication Set 2 were compared. ‘Correlation’ denotes the Pearson’s correlation coefficient with its significance denoted in the ‘p-value’ column. Rows denoted by ‘mean(PC1,PC2)’ indicate the correlation between the 50 genes with the largest average loading of PC1 and PC2.
