# Supplementary material for: Principal Component Analysis Characterizes Shared Pathogenetics from Genome-Wide Association Studies
Source: PLoS Comput Biol. 2014 Sep 11;10(9):e1003820. doi: 10.1371/journal.pcbi.1003820 (PMC4161298; doi:10.1371/journal.pcbi.1003820)
Supplement: Table S4 — Pathway enrichment after filtering nearby genes. Pathway enrichment was applied to a subset of genes that were located greater than 0.1 cM from each other. (DOC) [file pcbi.1003820.s017.doc]

| **PC** | **Pathway** | **FDR (q-value)** |
| --- | --- | --- |
| 1 | Intestinal immune network for IgA production | 0.028 |
|  | Antigen processing and presentation | 0.057 |
|  | Spliceosome | 0.141 |
|  | Inositol phosphate metabolism | 0.156 |
|  | Cell adhesion molecules | 0.19 |
|  | | |
| 2 | NOD-like receptor signaling pathway | 0.152 |
|  | GH pathway | 0.207 |
|  | Insulin pathway | 0.211 |
|  | CardiacEGF pathway | 0.213 |
|  | IL2 pathway | 0.226 |
|  | NFAT pathway | 0.236 |
|  | Dorso ventral axis formation | 0.236 |
|  | IL2RB pathway | 0.245 |
|  | IGF-1 pathway | 0.246 |

**Table S4. Pathway enrichment after filtering nearby genes.** Pathway enrichment was applied to a subset of genes that were located greater than 0.1cM from each other.
